# Combined action of albumin and heparin regulates lipoprotein lipase oligomerization, stability, and ligand interactions

**DOI:** 10.1371/journal.pone.0283358

**Published:** 2023-04-12

**Authors:** Robert Risti, Kathryn H. Gunn, Kristofer Hiis-Hommuk, Natjan-Naatan Seeba, Hamed Karimi, Ly Villo, Marko Vendelin, Saskia B. Neher, Aivar Lõokene

**Affiliations:** 1 Department of Chemistry and Biotechnology, Tallinn University of Technology, Tallinn, Estonia; 2 Department of Biochemistry and Biophysics, The University of North Carolina at Chapel Hill, Chapel Hill, North Carolina, United States of America; 3 Laboratory of Systems Biology, Department of Cybernetics, Tallinn University of Technology, Tallinn, Estonia; Consejo Superior de Investigaciones Cientificas, SPAIN

## Abstract

Lipoprotein lipase (LPL), a crucial enzyme in the intravascular hydrolysis of triglyceride-rich lipoproteins, is a potential drug target for the treatment of hypertriglyceridemia. The activity and stability of LPL are influenced by a complex ligand network. Previous studies performed in dilute solutions suggest that LPL can appear in various oligomeric states. However, it was not known how the physiological environment, that is blood plasma, affects the action of LPL. In the current study, we demonstrate that albumin, the major protein component in blood plasma, has a significant impact on LPL stability, oligomerization, and ligand interactions. The effects induced by albumin could not solely be reproduced by the macromolecular crowding effect. Stabilization, isothermal titration calorimetry, and surface plasmon resonance studies revealed that albumin binds to LPL with affinity sufficient to form a complex in both the interstitial space and the capillaries. Negative stain transmission electron microscopy and raster image correlation spectroscopy showed that albumin, like heparin, induced reversible oligomerization of LPL. However, the albumin induced oligomers were structurally different from heparin-induced filament-like LPL oligomers. An intriguing observation was that no oligomers of either type were formed in the simultaneous presence of albumin and heparin. Our data also suggested that the oligomer formation protected LPL from the inactivation by its physiological regulator angiopoietin-like protein 4. The concentration of LPL and its environment could influence whether LPL follows irreversible inactivation and aggregation or reversible LPL oligomer formation, which might affect interactions with various ligands and drugs. In conclusion, the interplay between albumin and heparin could provide a mechanism for ensuring the dissociation of heparan sulfate-bound LPL oligomers into active LPL upon secretion into the interstitial space.

## Introduction

Lipoprotein lipase (LPL) is a crucial enzyme in the intravascular hydrolysis of triglyceride-rich lipoproteins (TLRs). LPL deficiency causes hypertriglyceridemia, which is causally linked to cardiovascular disease (CVD) and pancreatitis[1–5]. LPL and its regulators are potential drug targets for the treatment of hypertriglyceridemia[6–8]. LPL is mainly produced by adipose and muscle parenchymal cells and secreted into the interstitial space where a capillary endothelial protein called glycosylphosphatidylinositol-anchored high-density lipoprotein-binding protein 1 (GPIHBP1) transports LPL to the endothelium of the vascular lumen[9, 10]. At this site, LPL catalyzes the hydrolytic degradation of triglycerides in chylomicrons (CM) and very low-density lipoproteins (VLDL). In addition to GPIHBP1 and lipoproteins, secreted LPL is known to interact with apolipoprotein CII[11], fatty acids[12, 13], heparan sulfates [14], and angiopoietin-like proteins (ANGPTLs) 3, 4, and 8 [15]. LPL activity is also indirectly affected by apolipoproteins CIII, CI [16], and AV [17]. Combined action of these ligands may play a key role in the extracellular regulation of LPL activity [17, 18]. Regulators and ligands can affect LPL in a variety of extracellular regions, including the interstitial space and vascular endothelium. In the postprandial state, LPL is also associated with circulating TRLs and is involved in the uptake of remnant lipoproteins by the liver [19].

Although significant progress has been made in understanding the complex regulation system of LPL, there are still several aspects that require further investigation. This is because the basic mechanistic knowledge about LPL and its regulators is primarily based on experiments performed with purified proteins and synthetic lipid substrates in conventional buffer solutions. However, it should be considered that in the native capillary plasma environment, which contains thousands of proteins and other molecules, extracellular effects on LPL can occur. The high plasma protein content (80 g/liter) is sufficient to produce a macromolecular crowding effect [20] that may affect LPL and its ligands. Many studies indicate that the stability, structure, and aggregation of proteins in general can be significantly affected by the macromolecular crowding effect [21–23]. In addition to the crowding effect, LPL may be affected by plasma components which have not been identified yet.

Numerous studies indicate that LPL can exist in different conformational and oligomeric states. It has been shown by several studies that purified and active LPL appears as a dimer. These dimers rapidly exchange subunits and can irreversibly dissociate into inactive monomers [24, 25]. According to this mechanism, a part of the active LPL pool is always in monomeric form. Recent studies have indicated that LPL can be in an active monomeric form under certain conditions [26]. In addition, based on recent x-ray crystal studies, monomeric LPL forms a stable 1:1 complex with its transporter GPIHBP1 [27, 28]. This state has been proposed to be the active form of LPL at the vascular endothelium [29]. Under physiological salt concentration, temperature and pH, isolated LPL is very unstable and loses most of its activity within minutes. This inactivation occurs due to conformational changes in the N-terminal domain of LPL and leads to the irreversible formation of inactive monomers that can form amorphous aggregates [25]. The inactive monomeric LPL has lower affinity for heparin than the active form, this difference is used for their separation using heparin-chromatography [30]. ANGPTL3, 4 and 8 actively regulate the ratio between inactive and active LPL as a means of post-translational regulation of LPL in tissues [31]. Similar to the thermal inactivation, ANGPTL4 induces conformational changes in the N-terminal domain of LPL [32]. However, the states of LPL are not limited to dimer and monomer, as recent data revealed that natively folded LPL can form helical oligomers at high concentrations, which are stabilized by the presence of heparin [33]. These oligomers are composed of dihedral LPL dimers, and unlike the amorphous irreversible aggregates of LPL, their dissociation restores the catalytic activity of LPL. The existence of inactive, helical form of LPL raises the question of under which conditions is LPL activity irreversibly vs. reversibly lost. In addition, there is the question of where different oligomeric forms of LPL are present in the body, and how LPL transitions between these forms.

LPL oligomerization varies as it travels through the cell, interstitial space, and vascular endothelium. Prior to secretion, it has been shown that LPL forms helical oligomers in the presence of heparan sulfate proteoglycans (HSPG) attached to syndecan-1 (SDC1) inside of adipose cell vesicles [33]. Following secretion, LPL has been found to linger at the cell surface in HeLa cells, tethered there by HSPGs [34]. This raises the question of what precipitates LPL dissociation from the HSPG, permitting LPL to transfer across the interstitial space and reach GPIHBP1. During this time in the interstitial space, it is crucial that LPL does not degrade prior to binding GPIHBP1, which facilitates LPL’s transfer into the capillaries. LPL is known to be stabilized by heparin, which is comparable to HSPGs [14, 33], which are present in the interstitial space. However, it begs the question whether there are additional components that influence LPL in the interstitial space, given the propensity of LPL to oligomerize in the presence of HSPGs.

In the present study we show that albumin, the major protein in blood plasma, forms a complex with LPL and has a significant effect on LPL stability and oligomerization. Surface plasmon resonance (SPR) measurements revealed that the LPL-albumin complex is dynamic, with a lifetime of less than few seconds. The equilibrium dissociation constant of the complex is 30–70 μM, which is comparable to the albumin concentration in the interstitial space [35], suggesting the physiological relevance of the LPL-albumin interaction. Negative stain transmission electron microscopy (nsTEM) and raster image correlation spectroscopy (RICS) measurements indicate that albumin, like heparin, induces reversible concentration dependent oligomerization of LPL. However, the oligomers decomposed when both heparin and albumin were present, indicating that albumin may play a role in liberating LPL from HSPGs allowing LPL to transit across the interstitial space. The reversible oligomerization also protected LPL from inactivation by its physiological regulator ANGPTL4. Based on these observations, we conclude that the role of albumin in the LPL system is more diverse than the previously known role of binding lipolysis-derived fatty acids in the capillaries.

## 2. Materials and methods

### 2.1 Reagents

Normolipidemic non-fasting plasma samples were purchased from Tallinn Blood Centrum, aliquoted and stored at -80°C. Triglyceride-rich lipoprotein fractions (CM/VLDL) were isolated from human plasma by density gradient ultracentrifugation. Goat serum as a source of apoC-II was obtained from Invitrogen (#10000C). Bovine LPL was purified from bovine milk [36] and dialyzed against 1 M NaCl and 20 mM NaH_2_PO_4_, pH 7.4 or 20 mM Bis-Tris, pH 6.5. Recombinant human LPL was purchased from Bio-Techne (#9888-LL) with a specific activity of >2,500 pmol/min/μg as determined with 4-nitrophenyl butyrate. Recombinant N-terminal domain of ANGPTL4 (nANGPTL4^26-184^) was expressed in *E*. *coli* and purified as previously described [37]. Full-length ANGPTL4 was purchased from BioVendor (#RD172073100-HEK). Human GPIHBP1 was purchased from Sino Biological (#15388-H02H). Both proteins were assessed by reducing and non-reducing SDS-PAGE prior to use. 1,2-Di-O-lauryl-rac-glycero-3-glutaric acid 6-methylresorufin ester (DGGR) (#30058) and bovine serum albumin (#A7906) were purchased from Sigma Aldrich. Na-deoxycholate (#218590250) and heparin (#411212500) were purchased from Acros Organics. Polyethylene glycol 6000 (PEG 6) was purchased from Alfa Aesar (#A17541) and dextran 40000 (dextran 40) from Sigma Aldrich (#31389). The amino coupling kit (containing N-hydroxysuccinimide, N-ethyl-N9 [(diethylamino)propyl]carbodiimide, 1M ethanolamine) and BIAcore sensor chips were purchased from GE Healthcare. Fluorescence reagent ATTO610-NHS ester was purchased from Merck (#93259). Lipoprotein free human plasma (LFHP) from a single donor was obtained by flotation ultracentrifugation at d = 1.215 g/ml [38] and dialyzed against 150 mM NaCl. Residual triglyceride and cholesterol concentrations in LFPH were respectively 40 μM and 10.34 μM.

### 2.2 Determination of LPL activity using isothermal titration calorimetry (ITC)

Catalytic activity of LPL was measured using an ITC assay that allows to determine LPL activity in complex substrate systems including undiluted human plasma [39]. The assay is based on the detection of changes in heat rate as a result of LPL catalyzed hydrolysis of lipids. The heat rate detected by ITC corresponds to the release of fatty acids and is related to the catalytic activity of LPL. There is a linear correlation between heat production and the number of fatty acids released. LPL activity is expressed as μJ/s (microjoule per second). The experiments in this study were performed on a MicroCal PEAQ-ITC (Malvern) as previously described [40]. In a standard measurement, the calorimetric cell (200 μl) was filled with a substrate mixture and the syringe (40 μl) contained 200 nM LPL in 150 mM NaCl, 20 mM HEPES, pH 7.4 buffer with 50 mg/ml BSA and 10 IU/ml heparin. The reference cell was filled with MilliQ water (200 μl). The stirring speed in the sample cell was set to 1000 rpm in all experiments. Automatic baseline stabilization took 5–12 min, after which a 0.4 μl LPL injection was made. Following that, a 5 μl LPL injection was made, which increased LPL concentration in the cell by 5 nM and the new baseline was measured for 3 min. All incubations and measurements were carried out at room temperature. The sample cell and syringe were washed with 10% Decon 90 and rinsed with MilliQ water after each experiment. The syringe was additionally rinsed with methanol.

### 2.3 Determination of LPL activity with DGGR

LPL activity was determined with fluorogenic substrate DGGR, using a spectrofluorophotometer (Shimadzu RF-5301 PC, Shimadzu Corporation, Japan). The reaction was followed for 3 minutes at 25°C, using an excitation wavelength of 572 nm and an emission wavelength of 605 nm. Samples were prepared by incubating 200 nM LPL at room temperature in a 150 mM NaCl, 20 mM HEPES, pH 7.4 buffer with various supplements or in LFHP. The incubation mixture was diluted to 10 nM LPL in a measurement buffer containing 150 mM NaCl, 20 mM HEPES, 10 IU/ml heparin, 24 μM DGGR and 0.5% Triton X-100. A 4 mM stock solution of DGGR was prepared in ethanol.

### 2.4 Determination of the affinity for the interaction of LPL-BSA using ITC

The binding affinity between bLPL and BSA was determined using MicroCal PEAQ-ITC. The calorimetric cell was filled with 1.695 μM bLPL and 10 IU/ml heparin in 20 mM HEPES, 150 mM NaCl, pH 7.4 buffer. The syringe contained 752 μM BSA in the same conditions and the reference cell was filled with degassed MilliQ water. Titration was carried out at 25°C with a reference power set to 41.9 μJ/s and at a stirring speed of 750 rpm. Binding affinity was estimated based on 38 sequential injections of BSA where each injection increased BSA concentration in the cell by 3.76 μM. A control experiment (dilution of BSA) was carried out in the same conditions but without bLPL in the measurement cell. Data analysis and K_D_ estimation was performed with MicroCal PEAQ-ITC Analysis Software (Malvern).

### 2.5 Stabilization measurements for estimation of affinity for the interaction of LPL-BSA

Human or bovine LPL (200 nM) was preincubated alone or with various concentrations of BSA at room temperature in a solution of 20 mM HEPES, 150 mM NaCl, pH 7.4. After 60 minutes, LPL activity was determined at 25°C using DGGR as a substrate. Stabilization effect of BSA was calculated by subtracting the LPL activity at 60 minutes from the initial LPL activity. The affinity (expressed as K_D_) of BSA for bLPL or hLPL was calculated by the equation:

$$v=\frac{V_{m}∙[BSA]}{K_{D}+[BSA]}+v_{0}$$ (1)

where v is the catalytic activity of hLPL or bLPL at any BSA concentration, [BSA]—BSA concentration, v_0_ –catalytic activity of bLPL or hLPL in the absence of BSA, V_m_−maximal bLPL or hLPL activity achievable by increasing the BSA concentration. To obtain estimation for K_D_, SigmaPlot software (SPSS, Chicago, IL, USA) and its hyperbolic curve fit function was used for fitting the experimental data according to Eq 1.

### 2.6 Surface plasmon resonance measurements

SPR experiments were performed on a Biacore 3000 instrument using CM5 sensorchips (GE Healthcare). The binding of BSA to bLPL was analyzed using two experimental set ups. In one experiment, biotinylated bLPL was attached to the surface of a CM5 sensor chip in 20 mM HEPES, 150 mM NaCl, pH 7.4 buffer via neutravidin that was covalently pre-immobilized using the amino coupling kit (GE Healthcare). It has been previously demonstrated that bLPL immobilized in this manner retains its ability to bind lipoproteins and ANGPTL4 [41]. 1074 RU of biotinylated bLPL was bound to the surface of the sensor which corresponded to a surface density of 19.5 fmol/mm2. In the other experiment, bLPL in 20 mM HEPES, 150 mM NaCl, 10 IU/ml heparin, 2 mg/ml BSA, pH 7.4 buffer was attached to GPIHBP1 that was covalently pre-immobilized in 10 mM acetic acid (pH 3.5) using the amino coupling kit. In this case, 215 RU of bLPL was bound to the immobilized GPIHBP1 corresponding to surface density of 3.9 fmol/mm2. Anti-LPL monoclonal antibody 5D2 was immobilized to a sensor chip according to Mysling et al. [42]. In this case, bLPL was bound to the immobilized 5D2 in 20 mM HEPES, 150 mM NaCl, 10 IU/ml heparin, pH 7.4. The surface density of bound LPL was 1.7 fmol/mm2. Binding of BSA to the sensor chips with bLPL were carried out at 25°C in a buffer that contained 20 mM HEPES, 150 mM NaCl, pH 7.4. For data analysis, nonspecific binding and bulk effect was subtracted from the total binding.

For calculation the number of BSA molecules bound to immobilized LPL (n), we used the following equation:

n=RUAMwARULMwL (2)

where RU_A_ is the response for binding of BSA to the immobilized bLPL at the steady state; RU_L_ is the amount of immobilized LPL expressed in response units; Mw_A_ and Mw_L_ are molecular weights of BSA (66 kDa) and bLPL (55 kDa), respectively.

### 2.7 Determination of diffusion coefficients using raster image correlation spectroscopy

LPL was labelled with ATTO610-NHS ester for these experiments. This LPL conjugate was prepared as follows: LPL was dialyzed against 200 mM NaHCO_3_, 1 M NaCl, pH 8.4 buffer, after which LPL was incubated with ATTO610 at a 1:5 molar ratio for 2 h at 4°C. The active fluorescent LPL was purified using heparin chromatography and the concentration and degree of labeling was determined by measuring optical densities at λ_280_ and λ_616_. Samples for RICS were prepared by incubating 10 nM LPL-ATTO610 with 190 nM unlabeled LPL at room temperature for up to 2 hours in buffer containing 20 mM HEPES pH 7.4, 150 mM NaCl and either 2 or 50 mg/ml BSA or 10 IU/ml heparin. Heparin was added after 15 minutes into LPL incubation with BSA in measurements with both BSA and heparin. LPL was incubated for at least 2 hours in incubations with 1 M GuHCl to allow for monomerization of LPL to happen [43].

Raster image correlation spectroscopy (RICS) was used to determine diffusion coefficient assuming that the sample consisted of a single component. Previously developed RICS extensions were used [44, 45]. Namely, sample was scanned at three different line scanning frequencies and imaging a region of 20 x 20μm. The signal was acquired during half of the scan time with the other half used for flyback with each image containing 1000 lines and pixel time set to 1μs. Images were acquired in sets of 90 (line scanning frequency 500 Hz), 60 (289 Hz), and 30 (167 Hz) frames with the order of the used frequencies randomized. Measurements were performed on custom built confocal microscope as previously described [45]. Analysis of the samples involved in this work required additional filtering step. Namely, samples contained a small fraction of large particles that were visible on some of the images. Such large particles disturbed the correlation analysis and were removed from overall diffusion coefficient determination, as described in Results. To filter the images with the large particles, each acquired image was split into four equal sections and correlation functions (CF) for each section was found. CF from each section was fitted by single-component diffusion model and the diffusion coefficient together with the number of particles in the confocal volume. For image sections with the large particles, estimated diffusion coefficient and number of particles in confocal volume were much smaller than for the image sections without such particles (see Results for examples). The sections without large particles were later analyzed by finding CF on the basis of all data and fitting CF obtained for each line scanning frequency with the same model parameters. The used models assumed Gaussian form of the point spread function and triplet state. Measurements and analysis of RICS data was done blindly with the samples prepared and enumerated by personnel not involved in the measurements and analysis of RICS data to avoid any bias.

### 2.8 Negative stain transmission electron microscopy (nsTEM)

Samples were prepared as described for RICS. Briefly, LPL was diluted to 200 nM in buffer containing 20 mM HEPES pH 7.4, 150 mM NaCl, and 2 mg/ml BSA (A7906, Sigma) or 10 IU/ml Heparin (H19, Fisher scientific) and incubated for 30 min. For samples with both BSA and heparin, heparin was added 15 min into incubation. For samples with both heparin and triton X-100, triton X-100 was added 15 min into incubation. Following incubation, 5 μl of each sample was applied to a glow discharged ultrathin carbon nsTEM grid (CF300_CU_UL, EMS). Grids were glow discharged with a PELCO easiGlow for 25 seconds to ensure hydrophilization of the grid carbon. Sample was incubated on the grid for 1 min followed by a wash with 100 μl of 20 mM HEPES pH 7.4 and 150 mM NaCl. The grid was then stained for 1 min with a filtered 2% uranyl acetate solution in 50% ethanol, blotted, and allowed to dry. Grids were imaged using a TECNAI T12 microscope equipped with a Gatan Rio camera. Images were collected using DigitalMicrograph and analyzed with ImageJ.

## 3. Results

### 3.1 Effect of BSA on LPL stability

**Fig 1:**
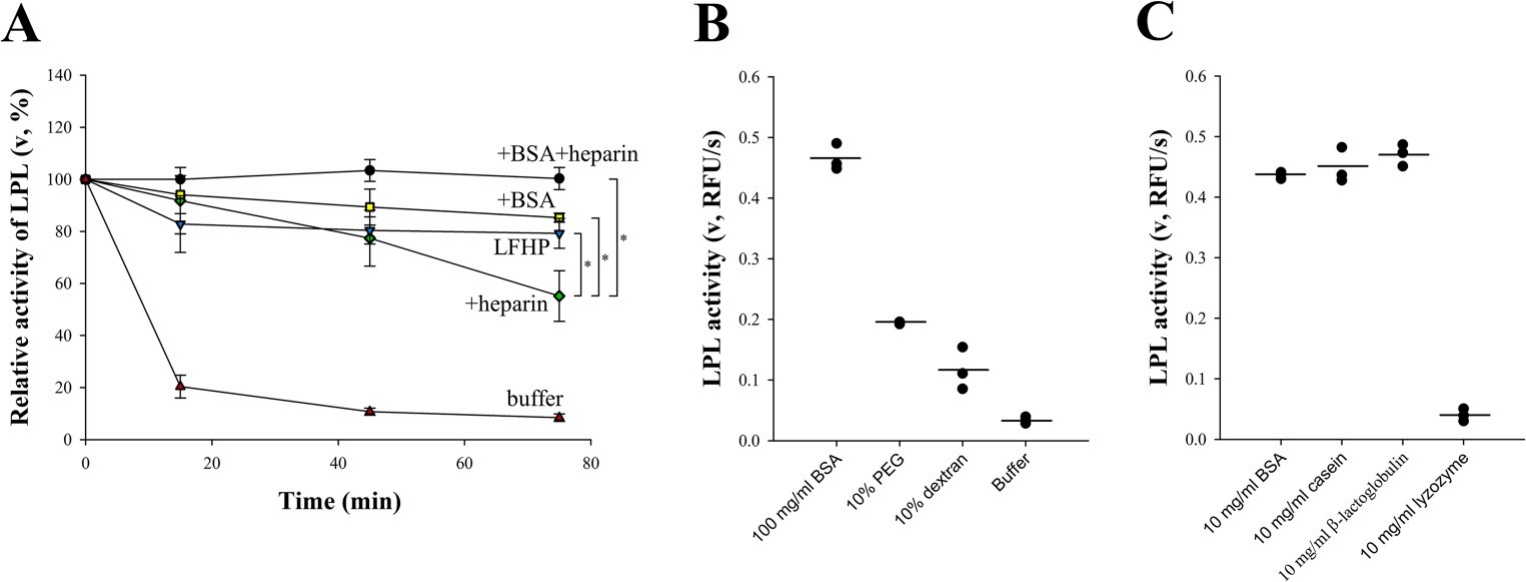
Catalytic activity of LPL measured with DGGR after incubation in various conditions. **(A)** 200 nM LPL was incubated in buffer A (▲), which contained 10 IU/ml heparin (◆), 50 mg/ml BSA (■) or both (●). Alternatively, LPL was incubated in LFHP (▼). LPL activity is expressed relative to the initial activity of the experiment that contained both heparin and BSA. Results at 75 minutes were compared by two-tailed Student’s *t*-test. *P<0.05. LPL loses its activity quickly in plain buffer but is stabilized by lipoprotein free human plasma (LFHP) or its main constituent albumin (BSA). The most significant stabilization was observed when both BSA and heparin were used together. **(B)** 200 nM LPL was incubated in macromolecularly crowded buffer A. LPL activity is expressed as relative fluorescence units per second (RFU/s). PEG 6 nor dextran 40 could stabilize LPL like BSA, indicating that macromolecular crowding alone is not sufficient for LPL stabilization. **(C)** 200 nM LPL was incubated in buffer A with various proteins. Casein and β-lactoglobulin were as efficient as BSA in stabilizing LPL, however lysozyme failed to exert any effect. This suggests that LPL stabilization by proteins depends on their specific characteristics such as charge or hydrophobicity.

LPL irreversibly loses most of its catalytic activity in ordinary buffers within minutes but is stable for several hours in undiluted human plasma as shown by the measurements of ITC [39]. This stabilization could be due to the binding of LPL to lipoprotein surfaces, as this effect has also been observed in some artificial substrate emulsions [46]. To examine whether other plasma components affect LPL stability, incubations were performed in lipoprotein free human plasma (LFHP), and in various other solutions for comparison: a) 150 mM NaCl, 20 mM HEPES, pH 7.4 buffer (buffer A), b) buffer A supplemented with BSA, the main plasma protein, c) buffer A supplemented with heparin (a well-known stabilizer of LPL), d) buffer A supplemented with both BSA and heparin. LPL activity was measured at multiple time-points using the fluorogenic substrate DGGR. As seen in **Fig 1A**, LPL lost almost 80% of its initial activity within 15 min in buffer A. Significant stabilization was recorded in all other solutions used. LFHP and BSA were somewhat more effective stabilizers than heparin. The most significant LPL stabilization was observed when both BSA and heparin were present, as LPL was completely stable for 75 minutes in this case. The similar stabilization effect of BSA and LFHP suggests that albumin is a major stabilizer of LPL in plasma.

We next wanted to address the mechanism by which BSA might stabilize LPL. We first investigated whether BSA was acting as a macromolecular crowder, effectively stabilizing LPL by altering the properties of the surrounding buffer environment. For this purpose, BSA was replaced with 10% PEG or 10% dextran in the LPL incubation mixture with buffer A. The concentration of 10% for PEG or dextran was chosen to produce a crowding effect that is comparable or higher than that of BSA at the concentration 100 mg/ml [47]. The results of these experiments are presented as residual LPL activity determined after 60 minutes of incubation (**Fig 1B**). The loss of LPL activity was greater in buffer conditions without crowders, but neither PEG nor dextran stabilized LPL to the extent BSA did. LPL activity was at least two-fold higher when incubated with BSA instead of PEG or dextran. This observation suggests that macromolecular crowding alone is not sufficient to cause the stabilization of LPL.

We did find that BSA was not the only protein that stabilized LPL as the milk proteins casein and β-lactoglobulin had a similar ability (**Fig 1C**). However, lysozyme, a small antibacterial protein, lacked this property. Thus, the stabilization of LPL seems not to be a general property of proteins, but rather depends on their specific characteristics. The effect of casein agrees with previous studies (casein stabilizes LPL in milk [48]) and β-lactoglobulin may also play a similar role. These proteins were chosen for comparison randomly based on their stability and solubility at room temperature.

**Fig 2:**
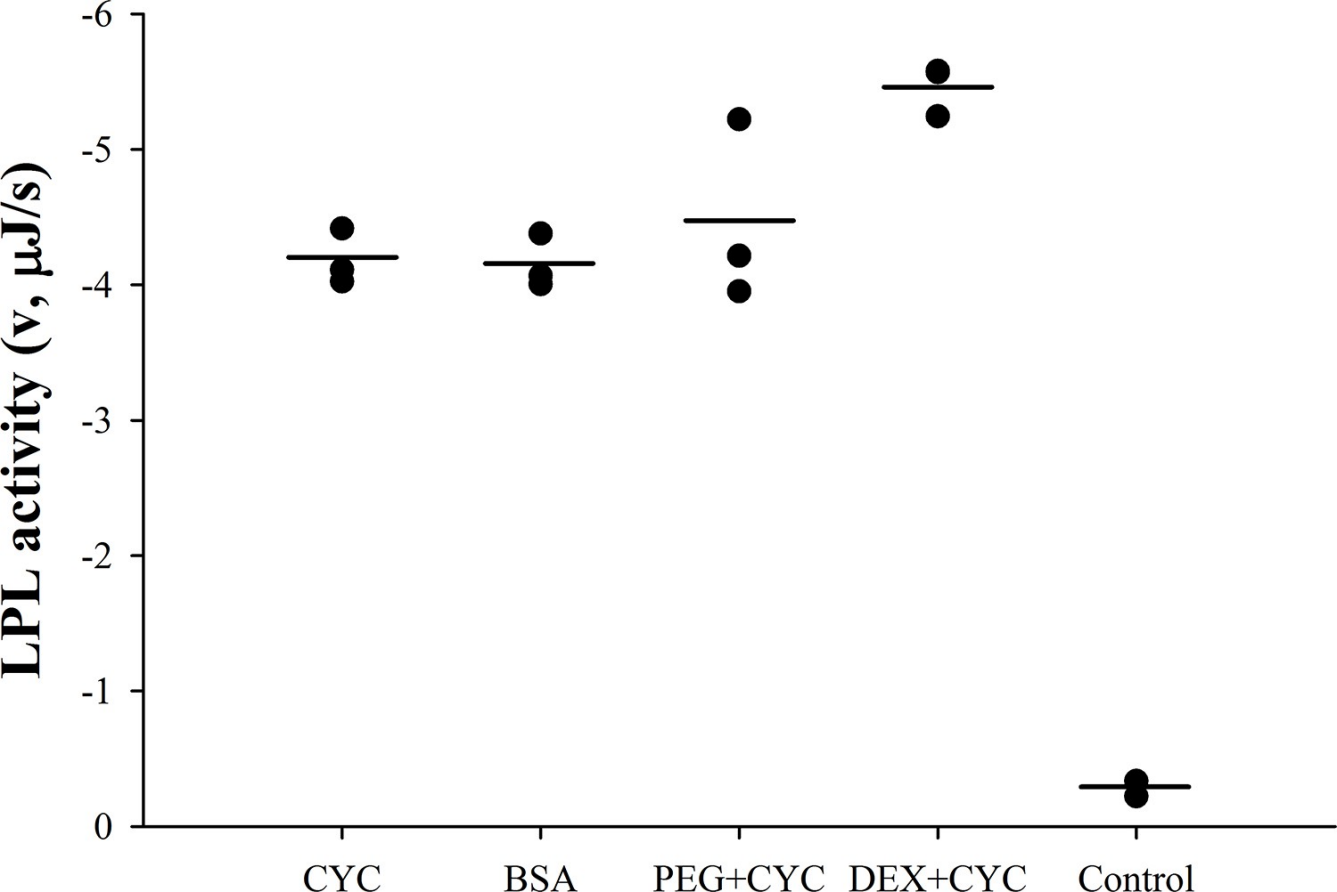
Catalytic activity of LPL on triglyceride-rich lipoproteins in the presence of fatty acid acceptors and macromolecular crowders. Measurements were performed using ITC. LPL activity is expressed as heat rate (μJ/s). The substrate mixture contained CM/VLDL (adjusted to 0.5 mM triglycerides), 50 mg/ml BSA (0.75 mM) or 10 mg/ml β-cyclodextrin (8.81 mM) (CYC) and either 10% PEG 6 or 10% dextran 40 (DEX).

The above experiments suggest that albumin plays a role in stabilizing LPL in the absence of substrate, the situation that occurs in vivo in the interstitial space after secretion of LPL from parenchymal cells. However, albumin is also required as a fatty acid acceptor to keep LPL active on triglycerides of long chain fatty acids. In vivo this occurs at the vascular endothelium when LPL binds lipoproteins and hydrolyzes their triglycerides. In the absence of albumin, long chain fatty acids inhibit LPL activity [12]. Here, we investigated whether the effect of albumin on LPL is limited to the fatty acid binding during lipolysis of lipoproteins by replacing BSA with another fatty acid acceptor, β-cyclodextrin, in the calorimetric assay (**Fig 2**). LPL activity was determined with ITC using a mixture of isolated triglyceride-rich lipoproteins CM/VLDL (adjusted to 0.5 mM triglycerides) accompanied by BSA, PEG or dextran. The concentrations of BSA (0.75 mM) and β-cyclodextrin (8.81 mM) were chosen to be well above the required limit to bind all FFAs released during lipolysis. The lack of difference in LPL activity between the use of two different FFA acceptors, BSA and β-cyclodextrin, allows to conclude that the role of albumin during endothelial lipolysis is confined to binding released FFAs. Introducing a crowded environment did not seem to significantly affect LPL activity towards TRLs. Only a 20% increase in LPL activity was noticed when dextran was used as a macromolecular crowder, and no change was detected when BSA or PEG was involved. This implies that the role of albumin in stabilizing LPL is likely to occur prior to its movement onto the endothelial surface.

### 3.2 LPL forms a complex with BSA

**Fig 3:**
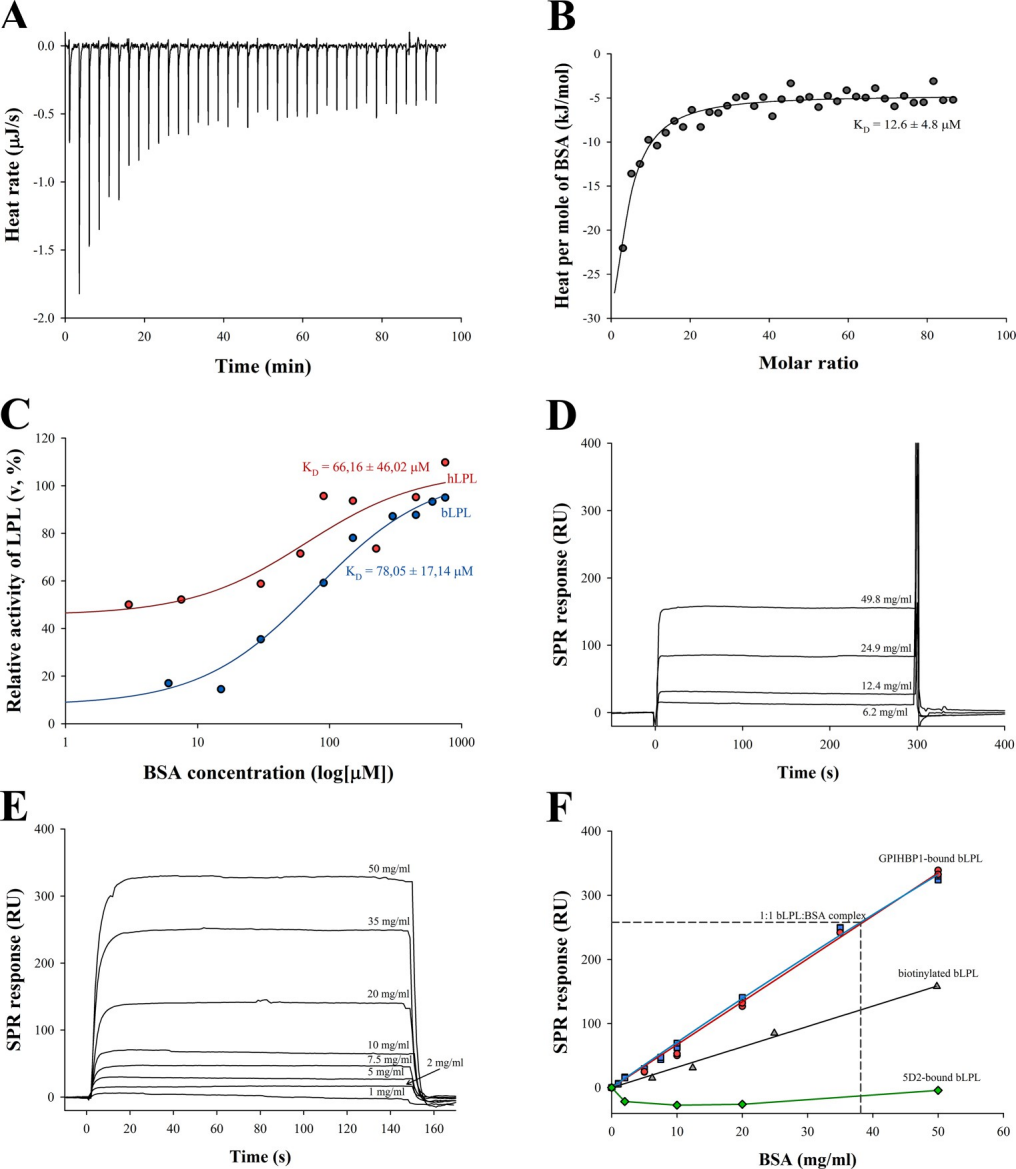
Interaction of LPL with BSA as studied using ITC, stabilization of LPL, and SPR. **(A)** An example ITC thermogram for the titration of BSA into a solution with bLPL, obtained after subtraction of the BSA dilution effect. The concentration of bLPL in the cell was 1.695 μM. **(B)** Fitted isotherm for the binding between BSA and bLPL from the ITC experiment on panel A. **(C)** Enzymatic stability of LPL in the presence of BSA as determined with DGGR. 200 nM bLPL or hLPL was incubated for 60 minutes in buffer A at various BSA concentrations. The values are calculated relative to the initial activity of LPL in the same conditions. BSA stabilized both bLPL and hLPL in a concentration-dependent manner. **(D)** SPR sensograms showing binding of BSA to biotinylated bLPL that was attached to pre-immobilized neutravidin. **(E)** SPR sensorgrams showing BSA binding to bLPL that was attached to pre-immobilized GPIHBP1. In D and E, BSA concentrations are shown on sensograms. Non-specific binding sensorgrams of BSA to streptavidin and GPIHBP1, respectively, have been subtracted. **(F)** Plateau values of sensorgrams plotted against BSA concentration. ●—Binding of BSA with 0.1 IU/ml heparin to GPIHBP1-bound bLPL. ▲—Binding of BSA to biotinylated bLPL. ◆—Binding of BSA to 5D2-bound bLPL. Dashed line—1:1 ratio of immobilized bLPL to bound BSA in the experiment with GPIHBP1-bound bLPL. The results indicate that BSA can bind to neutravidin-bound LPL or GPIHBP1-bound LPL but not 5D2-bound LPL.

Next, we investigated whether LPL was able to form a direct complex with BSA. The formation of the bLPL-BSA complex was first demonstrated by ITC by titrating BSA into a solution with bLPL (**Fig 3A and 3B**). This experiment was performed in the presence of heparin to stabilize bLPL. The bLPL-BSA interaction was exothermic and BSA concentrations above 3 μM were required to record a measurable heat effect. The low solubility of bLPL, the weakness of the interaction, and the solubility limit of BSA did not allow us to perform experiments under optimal conditions where all the parameters characterizing the interaction could be determined. Therefore, the ITC titration data could only be used to estimate the apparent affinity of the BSA-bLPL interaction. These data suggested a complex type of interaction: after an initial saturable binding phase, additional and almost constant heat production was recorded at higher BSA concentrations. The second weaker binding event was not well-defined to allow for the estimation of K_D_. When the second weaker binding phase was not taken into account, the calculated apparent K_D_ for the initial binding was 12.6 μM. However, our various simulations involving both phases confirmed that any additional binding has a negligible effect on the K_D_ of initial binding.

We also investigated the interaction between LPL and BSA by measuring the enzymatic stability of bovine LPL (bLPL) and human LPL (hLPL) at different BSA concentrations (**Fig 3C**). BSA stabilized both LPL homologs in a concentration-dependent manner and the stabilization effect was already detectable when BSA concentration was as low as 6 μM (0.4 mg/ml). A hyperbolic relationship was observed between the relative increase of bLPL or hLPL activity and BSA concentration. The calculated apparent K_D_ for the interaction between bLPL-BSA and hLPL-BSA were 78 μM (5.2 mg/ml) and 66 μM (4.4 mg/ml), respectively. The reason for the difference in the K_D_ values between the ITC and the stabilization study is likely due to the presence of heparin in the ITC study.

In addition to ITC and enzymatic stability measurements, the interaction between bLPL and BSA was investigated by SPR. Three separate BSA binding experiments were performed using a Biacore 3000 (GE Healthcare): 1) with biotinylated bLPL non-covalently attached to neutravidin that was directly pre-immobilized on the sensor chip 2) with bLPL non-covalently attached to the chip surface via pre-immobilized GPIHBP1 3) with bLPL non-covalently attached to pre-immobilized 5D2 antibody. In all systems, dissociation of bLPL from the sensor chip was negligible. In the experiments with the neutravidin-bound biotinylated bLPL, a detectable association of BSA was observed when its concentration was above 6 mg/ml (91 μM) (**Fig 3D**) while in the case of GPIHBP1-bound bLPL, a detectable binding of BSA was observed at just 1 mg/ml (**Fig 3E**). In the case of 5D2-bound bLPL, at lower BSA concentrations of 5–20 mg/ml, the non-specific binding to 5D2 was higher than the binding to bLPL-5D2, however, minor association was observed when BSA concentration was as high as 50 mg/ml. We conclude that 5D2 strongly hinders association of BSA with LPL. The rectangular shape of the sensorgrams of the systems with GPIHBP1 and neutravidin indicated very fast off-kinetics, BSA was completely dissociated within few seconds. Such rapid dissociation made the correct calculation of rate constants impossible. The rapid dissociation kinetics also indicated that the lifetime of the bLPL-BSA complex is very short, less than a few seconds. For estimation of the equilibrium dissociation constant K_D_, the plateau values were plotted against the BSA concentrations in the injected solutions (**Fig 3F**). In both systems, this relationship remained linear over the range of BSA concentrations used (0–910 μM), meaning that no saturation was achieved. Higher BSA concentrations were not possible with the BIAcore system. Additionally, the presence of 0.1 IU/ml heparin did not affect the binding of BSA to GPIHBP1-bound bLPL (**Fig 3F**, red circles). Although the apparent binding kinetics of BSA to neutravidin-bound LPL and to GPIHBP1-bound LPL were similar, much less of the GPIHBP1-bound LPL than the neutravidin-bound LPL was required for detectable BSA binding. The surface density 3.9 fmol/mm^2^ of the GPIHBP1 bound LPL was sufficient to observe the interaction while a measurable association was detected when the surface density of neutravidin-bound biotin-bLPL was 19.5 fmol/mm^2^. When measuring 50 mg/ml BSA binding, the steady-state bound BSA/LPL molar ratio was 0.12:1 for neutravidin-bound LPL and 1.2:1 for GPIHBP1-bound LPL. This indicates that the main part of the neutravidin-bound LPL was not able to interact with BSA. At the same time, a significant fraction, if not all, of the LPL bound to GPIHBP1 interacted with BSA. Non-saturable linear binding, as well as a greater than one molar ratio of bound BSA/LPL, suggest that each molecule of LPL attached to GPIHBP1 can simultaneously interact with multiple BSA molecules. In summary, all three different binding studies confirm that LPL forms a complex with BSA at physiologically relevant concentrations, as circulating albumin concentrations range from 35 to 50 mg/ml and 4.4–15.7 mg/ml in the interstitial space, the latter depending on the tissue [35].

### 3.3 Interplay between albumin and heparin differs from their individual effects

**Fig 4:**
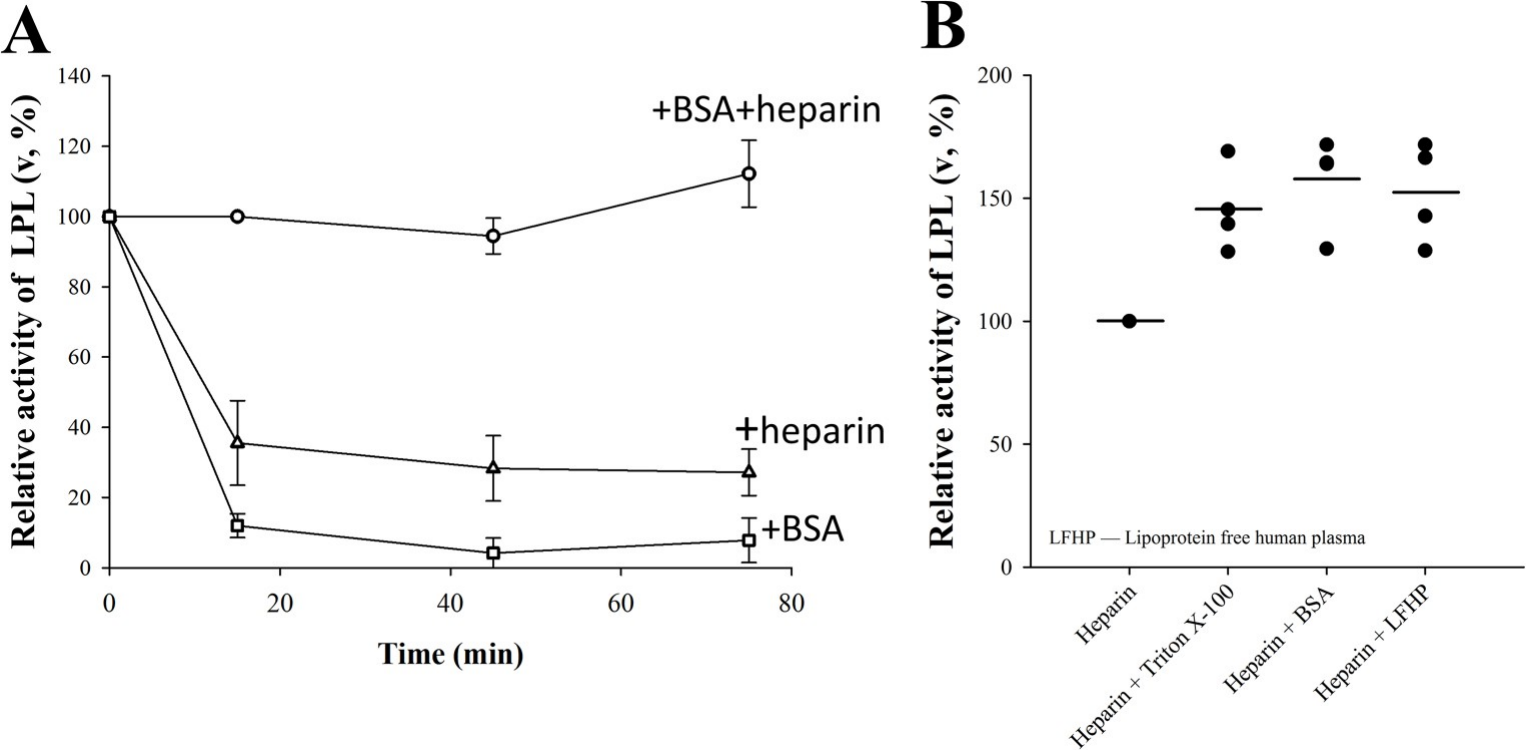
Effect of BSA, Triton X-100 or LFHP on LPL activity in the presence of heparin. **(A)** 200 nM LPL was incubated for the indicated timepoints with 50 mg/ml BSA, 10 IU/ml heparin or both in buffer A. The remaining activity (expressed as heat rate, μJ/s) was determined with ITC after a single 5 nM LPL injection into human plasma that contained 1.31 mM triglycerides. LPL activity is expressed relative to the initial activity of the experiment that contained both heparin and BSA and the data is presented as mean ± SD of three independent measurements. LPL activity was significantly lower when only BSA or heparin was used. **(B)** 1 μM LPL was incubated with 10 IU/ml heparin in buffer A and diluted 5-fold to buffer A with heparin and 0.5% Triton X-100, 50 mg/ml BSA or LFHP. The remaining LPL activity was determined in the same manner as panel A. LPL activity was restored equally with every additive.

Building on the data that LPL forms a complex with BSA and previous works showing that LPL interacts with heparin, we wanted to investigate the independent and combined effects of BSA and heparin on LPL when LPL activity is determined with a natural substrate. When 200 nM LPL was incubated for 15 minutes with BSA or heparin alone, its activity on TRLs in human plasma, as measured by ITC, was much lower than when incubated in the presence of both heparin and BSA (**Fig 4A**). Our data therefore shows that when acting on a natural substrate, both heparin and BSA are needed during incubation for maximum LPL activity. This difference was not observed in the DGGR assay (**Fig 1A**) under the same incubation conditions, nor when LPL activity was measured using the tributyrin/gum arabic substrate system (**S1 Fig**). To elucidate the reason for this discrepancy between the assays, effects of various incubation conditions were tested. We found that LPL, which has been incubated with heparin alone for 15 minutes regains its maximal activity after dilution to a solution containing BSA (**Fig 4B**). A similar increase in LPL activity was also observed when the dilution was carried out into LFHP or Triton X-100 (**Fig 4B**). These data suggest that the low activity of LPL measured in human plasma when preincubated with heparin (**Fig 4A**) was due to reversible changes in the state of LPL. It has recently been shown that heparin induces formation of reversible inactive helical LPL oligomers that dissociate into active LPL in the presence of the surfactant deoxycholate [33]. Our data here indicate that the LPL helical oligomers do not dissociate into the active form in undiluted human plasma but do so in the substrate systems containing surfactants such as Triton X-100 in the DGGR assay or gum arabic in the tributyrin assay. The LPL helical oligomers also dissociated in the substrate-free preincubation solutions with Triton X-100, BSA, or LFHP. The observation that the oligomers did not dissolve in undiluted human plasma but did when lipoproteins were removed from plasma—that is, in LFHP,—suggests that the presence of lipoproteins hindered this process.

**Fig 5:**
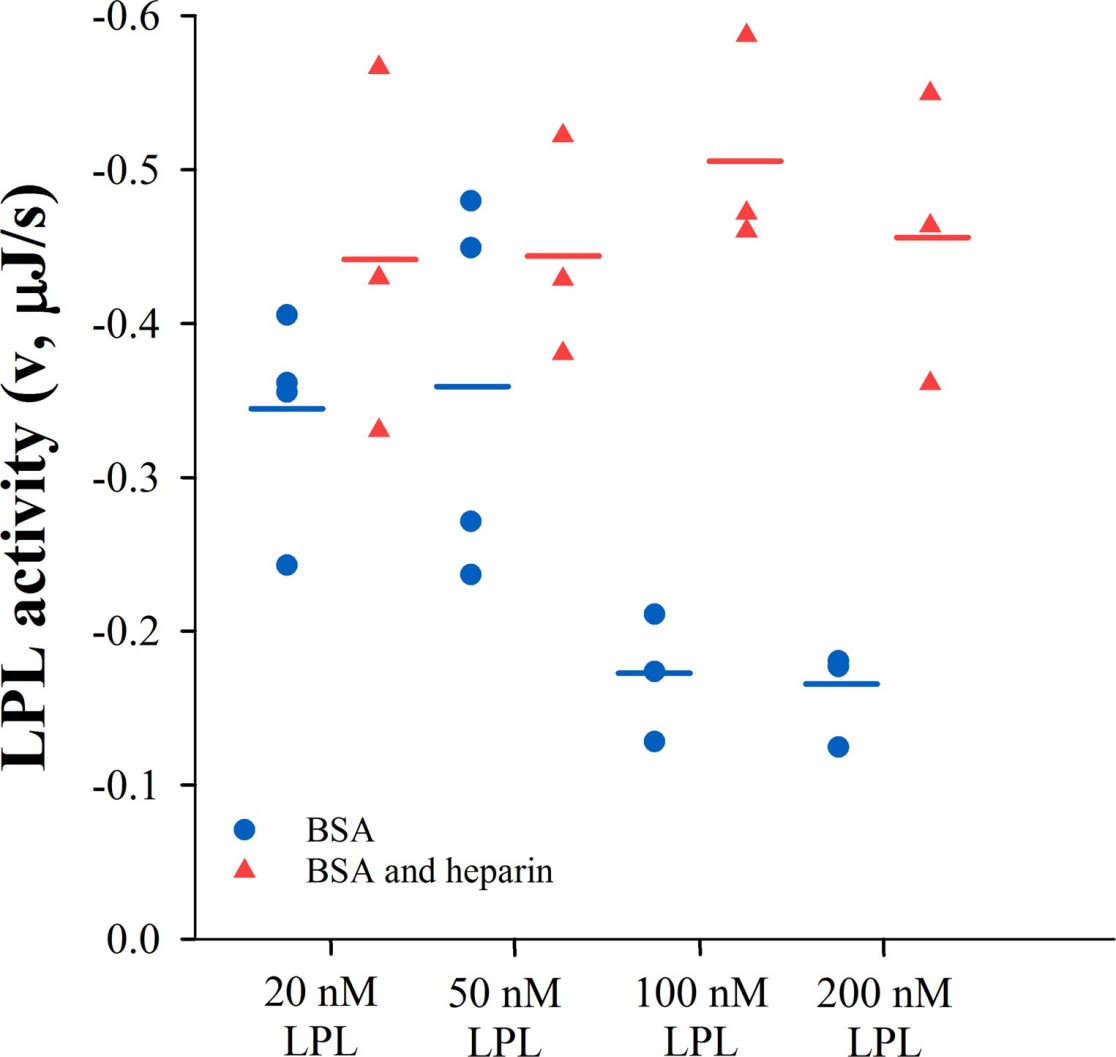
Effect of LPL incubation concentration on its activity as measured with ITC. LPL activity, expressed as heat rate (μJ/s), after incubation of LPL at various concentrations with 50 mg/ml BSA with (▲) or without (●) 10 IU/ml heparin in buffer A. The changes in residual LPL activity suggest that LPL oligomerization triggered by BSA is dependent on LPL concentration. This dependence disappears with the combined use of heparin and BSA which blocks the formation of LPL oligomers.

When LPL at various concentrations was preincubated with BSA alone prior to activity determination in human plasma by ITC, the observed catalytic activity of LPL was higher at its lower preincubation concentrations. A significant increase in the activity of LPL was observed when preincubation concentration was reduced from 200 nM to 20 nM (**Fig 5**). This sharp change in LPL activity suggests that BSA, like heparin, causes reversible oligomerization of LPL. Like heparin induced LPL oligomers, BSA induced LPL oligomers did not dissociate into active form in human plasma. The LPL concentration effect on its activity disappeared when heparin was combined with BSA during preincubation. In this case, LPL activity was the same for every concentration (**Fig 5**).

### 3.4 Oligomerization of LPL as studied by RICS and Transmission Electron Microscopy (TEM)

**Fig 6:**
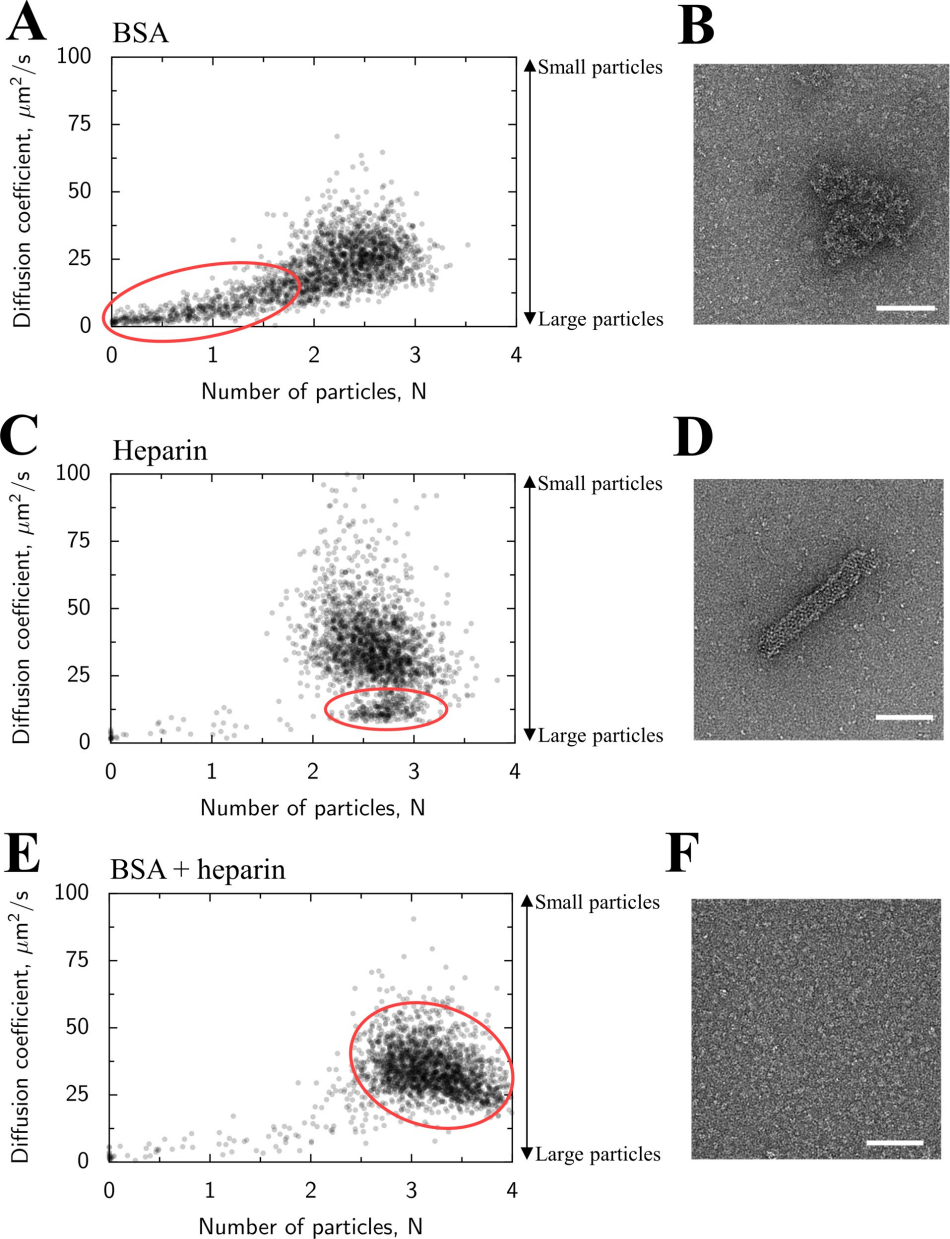
Oligomerization of LPL as studied by RICS and TEM. 10 nM LPL-ATTO610 with 190 nM unlabeled LPL was incubated in buffer A with the indicated supplements for up to two hours at room temperature. **(A)** Relationship between diffusion coefficient (D) and number of particles (N) in confocal volume (proportional to concentration) for image sectors recorded in an experiment with 200 nM LPL and 50 mg/ml BSA. Larger particles have lower D values. Notice that a large fraction of measurements leads to estimates with low D and N values (red circle) **(B), (D), (F)** Representative nsTEM micrographs of unlabeled LPL in similar conditions as RICS experiments. Scale bars are 100 nm. **(C)** Interaction of LPL and heparin leads to formation of a fraction of estimates with lower D values at N values between 2–3. This is visible in the plot as a smaller set of points grouped around D = 10 μm^2^/s (red circle) with the larger set of estimates above it. **(E)** Relationship between D and N for an experiment with LPL, BSA and heparin. Notice that most of the image sectors lead to D and N estimates in a certain region of the plot (red circle).

To further characterize the LPL oligomerization occurring in the presence of heparin and BSA, we used RICS technology to determine diffusion coefficients (D) and estimate the relative content of LPL species with different sizes. Larger particles will have a lower D value while smaller particles are characterized by high D values. We chose RICS technology instead of the previously used standard sedimentation method [14, 26] because the high sucrose concentration used in the latter would cause additional crowding effect [49, 50]. In the RICS experiments, LPL was labelled with the fluorescent marker ATTO610, and measurements were performed in the presence of either BSA, heparin, or both. No fluorescence correlation was observed in measurements performed with BSA alone in solution. Straightforward fitting of autocorrelation functions, as a part of RICS analysis, produced poor fits (results not shown). This was caused by the presence of a population of large bright particles which disturbed autocorrelation significantly and made it impossible to fit with the diffusion models. To resolve this issue, the recorded images were split into subsections and the diffusion coefficient with the number of particles (N) in the confocal volume was estimated for each of these subsections. As it is shown in **S2A Fig**, the estimated D varied from subsection to subsection throughout the experiment, including some with relatively small values. When plotting D against N, one can observe that there is a fraction of subsections with small D that have small N (bottom left corner of **Fig 6A, 6C and 6E**). This is consistent with large bright particles moving through the experimental area–leading to low D and small N values. The high variability of D and N estimates is expected as we used small datasets to make those estimations. Subsections with the small estimated D and N were removed and the average correlation functions for the remaining subsections were found and fitted with the diffusion model (**S2B Fig**). Analysis of the LPL diffusion coefficients for different samples is summarized in **Table 1**, the average diffusion coefficient takes into account all observed larger and smaller species. RICS experiments were conducted using both 200 nM and 10 nM LPL to observe differences in the average diffusion coefficients resulting from LPL concentration.

**Table 1:** Calculated diffusion coefficients of main LPL component from RICS measurements.

LPL	BSA	10 IU/ml heparin	Diffusion coefficient (D, μm^2^/s)
200 nM	50 mg/ml		7.0 ± 1.2
	2 mg/ml		13.0 ± 2.9
		➕	19.9 ± 3.4
	50 mg/ml	➕	21.7 ± 0.8
	2 mg/ml	➕	25.7 ± 2.6
10 nM	50 mg/ml		20.5 ± 1.4
	2 mg/ml		28.7 ± 2.2
	50 mg/ml	➕	24.0 ± 1.0
10 nM LPL + 1 M GuHCl			42.5 ± 7.2

LPL at 200 nM formed larger particles in the presence of 50 mg/ml BSA, as indicated by a low average D (7.4 ± 1.2 μm^2^/s). In addition, as shown in **Fig 6A**, the sample was heterogeneous, leading to formation of tail-like structure on N-D scatter plot (red circle). We predict that the large particles represent the reversible oligomerized state of LPL identified with BSA. Oligomerization of LPL was also seen with 10 IU/ml heparin (**Fig 6C**), albeit in a smaller proportion as indicated by larger average D of ~20 μm^2^/s (**Table 1**). Similar average D (~22 μm^2^/s) was recorded after addition of 10 IU/ml heparin to the LPL+BSA mixture homogenized the LPL distribution (**Fig 6E**). This agrees with ITC measurements that show LPL activity of ATTO610 labeled LPL is restored after addition of heparin to an LPL-BSA incubation (**S3 Fig**), similarly to how BSA was previously able to restore LPL activity in a heparin containing solution (**Fig 4B)**. The same pattern was observed at 2 mg/ml BSA concentration (**Table 1**). However, on the basis of D measurements, it is impossible to explain the differences in activity of LPL with heparin alone and BSA with heparin. In solution, LPL is likely in a similar size, but inactive conformation, in the presence of heparin at this LPL concentration, as indicated by activity measurements.

TEM experiments were used to examine the appearance of LPL aggregates under the conditions used in the RICS measurements, but without ATTO610 labeled LPL. LPL aggregates were observed with BSA (**Fig 6B**), which were not seen with BSA alone (**S4A Fig**). LPL helical oligomers were observed when incubated with heparin (**Fig 6D**). LPL aggregates were not observed when combined with both BSA and heparin (**Fig 6F**), although combining heparin and BSA did lead to some aggregation of the BSA (**S4B Fig**). The data from RICS supports that these BSA aggregates in the presence of heparin do not contain LPL. LPL aggregates were also observed with 200 nM LPL by itself (**S4D Fig**). Finally, LPL oligomers in the presence of heparin were dissolved by treatment with Triton X-100 (**S5 Fig**), which is in agreement with LPL activity measurements that show an increase in LPL activity after introduction of Triton X-100 to oligomeric LPL conditions (**Fig 4B**).

To test the effect of LPL concentration on formation of LPL oligomers, measurements were performed with 10 nM LPL and 50 mg/ml BSA, in the absence or presence of 10 IU/ml heparin. Measurements with 10 nM LPL and 50 mg/ml BSA exhibited only slight oligomerization (**Table 1**, average D ~20 μm^2^/s) and the results were comparable to 10 nM LPL with BSA and heparin together. This agrees with the concentration dependence demonstrated in **Fig 5A**. Finally, 10 nM LPL was denatured with 1 M GuHCl to produce partially unfolded monomeric LPL [43] as a comparison (**S2C Fig**). In this case, the LPL solution is inhabited by smaller particles whose average Ds were approximately twice as high as that observed when LPL was incubated with heparin and BSA.

### 3.5 Oligomeric form of LPL is more resistant to inactivation by ANGPTL4

**Fig 7:**
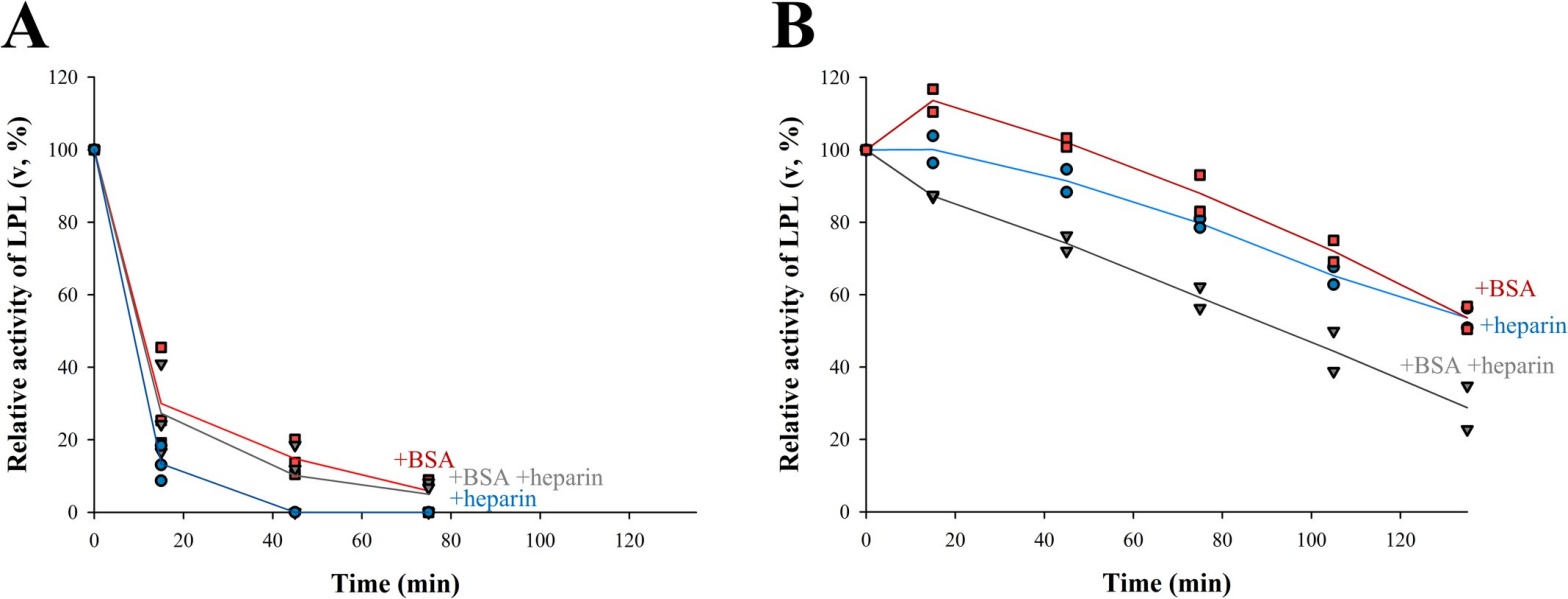
Inactivation of oligomeric LPL by ANGPTL4. 20 nM LPL **(A)** or 200 nM LPL **(B)** was incubated in the presence of 1 μM ANGPTL4 and 10 IU/ml heparin (●) or 50 mg/ml BSA (■) or both (▼) in buffer A. Remaining LPL activity was determined by ITC using undiluted pooled human plasma (0.89 mM triglycerides, n = 5). LPL activity is expressed relative to initial activity in the same conditions without ANGPTL4. Symbols represent individual measurements and lines correspond to their average values. The results suggest that the rate of LPL inactivation by ANGPTL4 depends on the degree of LPL oligomerization.

We next asked if ANGPTL4 inactivation of LPL could be influenced by the formation of the LPL-BSA and LPL-heparin oligomers. At a relatively low LPL concentration of 20 nM where we do not expect to see significant oligomerization, we found the rate of inactivation by ANGPTL4 was similar in all conditions (**Fig 7A**). When the LPL concentration was raised to 200 nM (**Fig 7B**), the difference between the incubation conditions was noticeable—after 130 minutes of incubation, the residual LPL activity was 55% of the initial activity in the presence of heparin or BSA alone and 35% in the presence of both. These results indicate that conditions that promote oligomer formation, such as higher LPL concentrations and the presence of either BSA or heparin alone, reduce the rate of ANGPTL4 inactivation. Thus, it is reasonable to assume that LPL oligomers are resistant or less affected by the action of ANGPTL4. LPL was not fully resistant to inactivation by ANGPTL4, likely due to our previous observation that LPL is not entirely oligomeric in these conditions. Some fraction of total LPL remains catalytically active in these cases as seen in **Figs 4** and **5A**.

### 3.6 Albumin amplifies the inactivation of LPL by nANGPTL4 in the presence of substrate

**Fig 8:**
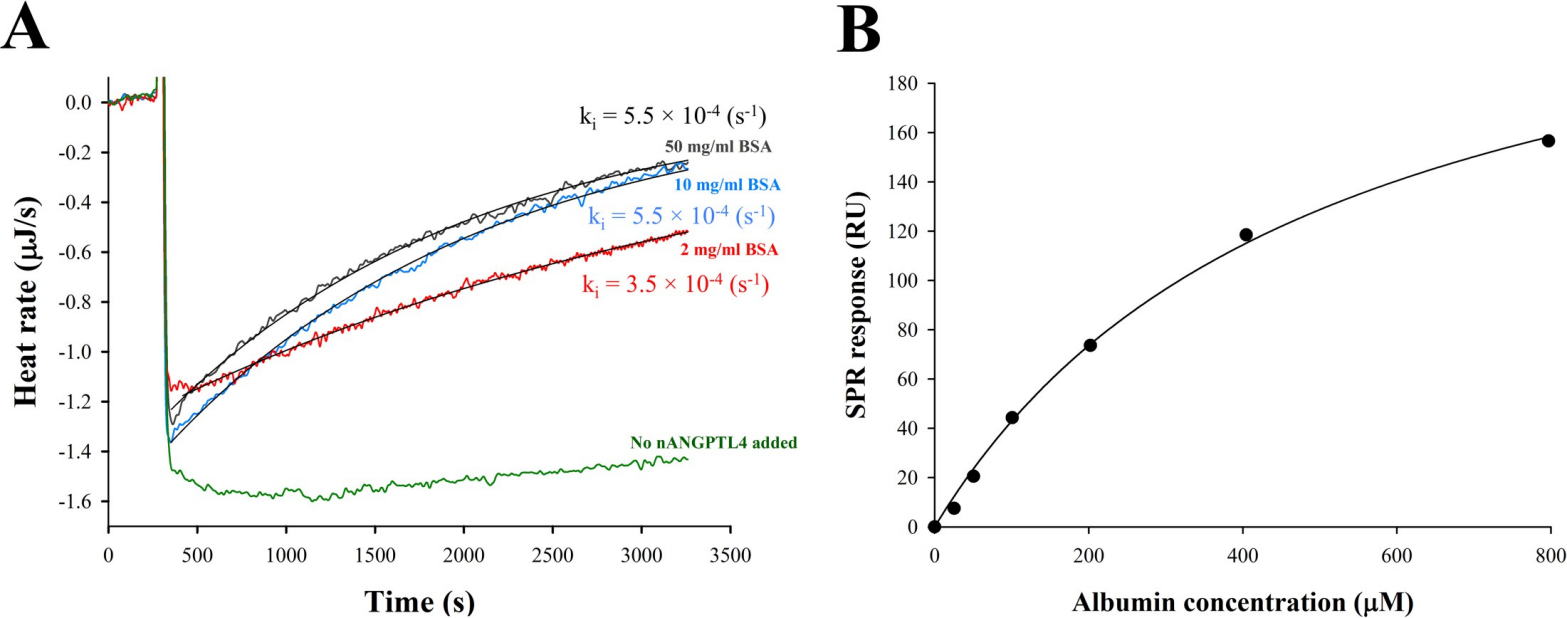
Interaction of albumin with nANGPTL4. **(A)** Effect of BSA at various concentrations on nANGPTL4-induced inactivation of LPL during lipolysis of triglyceride-rich lipoproteins. Raw ITC thermograms of lipolysis of CM/VLDL (adjusted to 0.75 mM triglycerides) by LPL in the presence of 100 nM nANGPTL4 and various concentrations of BSA (2 mg/l, 10 mg/ml, 50 mg/ml). The lipolysis rate is expressed as heat rate, μJ/s. The control experiment was performed in the absence of nANGPTL4 and presence of 50 mg/ml BSA. k_i_ represents inhibition rate constant of LPL by nANGPTL4 calculated from the data (n = 2). The results demonstrate that the rate of LPL inactivation by nANGPTL4 is increased at higher BSA concentrations. (**B)** SPR analysis of binding of various concentrations of HSA to immobilized nANGPTL4. Plateau values of SPR sensorgrams were plotted against HSA concentrations after subtracting non-specific binding. The results indicate that HSA interacts with nANGPTL4.

In addition to the interstitial space, ANGPTL4 can affect LPL at the surface of lipoproteins [51].To investigate whether albumin affects the ANGPTL4/LPL interaction in this case, inactivation kinetics of LPL by ANGPTL4 were measured at various BSA concentrations. These measurements were performed by ITC using a mixture of triglyceride-rich lipoproteins as substrate. We found that the rate of inactivation of LPL by nANGPTL4 was increased at higher BSA concentrations (**Fig 8A**). The inactivation curves followed the first-order kinetics and the inactivation rate constant (k_i_) in the presence of 10 mg/ml or 50 mg/ml BSA was about 1.6 time higher than for 2 mg/ml BSA. Since albumin does not affect LPL activity and stability at substrate surfaces, we wondered if there might be a direct interaction between albumin and nANGPTL4. Indeed, SPR measurements showed association between human serum albumin (HSA) and immobilized nANGPTL4 (**Fig 8B**). This interaction was rather weak (K_D_ = 500±51 μM), but still sufficient to have physiological significance in the capillary.

## Discussion

In the present study, we show that albumin, the major protein component of plasma, increases the stability of LPL via reversible oligomerization, dissolves heparin-induced LPL oligomers, and can affect the LPL-ANGPTL4 interaction. Thus, our results suggest that the role of albumin in the LPL system is more diverse than the previously known role of binding lipolysis-derived fatty acids. Albumin, usually as BSA, has been also previously included in LPL assays [52–54] but its specific role has never been studied before this study. Although most of the experiments in this study were performed with bovine LPL and BSA, it is likely that the results of the proteins from other sources will not differ because our experiments with human LPL and BSA did not differ significantly from those with bovine LPL. Also, LFHP, which contains HSA, affected LPL stability similarly to BSA. LPL is exposed to albumin and other plasma components in the vascular endothelium, interstitial space, the surface of parenchymal cells, and when attached to lipoprotein remnants [55]. The plasma concentration of albumin is 35–50 mg/ml and its concentration in the interstitial space of adipose tissue and skeletal muscle is 4.4–10 mg/ml and 9.7–15.7 mg/ml, respectively [35]. Since the K_D_ values of the LPL-albumin complexes were lower than or comparable to albumin concentrations in all extracellular locations of LPL, it is reasonable to assume that albumin affects LPL *in vivo*. Our data also indicate that albumin affects LPL stability via direct interaction, and macromolecular crowding may have only a limited role in the effect. The short lifetime of the LPL-albumin complex, as seen in the SPR experiment, may be important for the rapid transition of LPL to other complexes in the interstitial space to enable efficient shuttling of LPL to the vascular endothelium.

The observation that casein and β-lactoglobulin also stabilize LPL, but lysozyme does not, raises the question of what properties are required for a protein to interact with LPL. Our measurements were performed at pH 7.4, where albumin, casein and β-lactoglobulin are negatively charged due to their low pI, while lysozyme with its very high pI (11.1) is positively charged. The overall positive charge of LPL may explain its interaction with negatively charged albumin, casein and β-lactoglobulin, and also the avoidance of positively charged lysozyme. Exposed hydrophobicity, which plays a role in the thermal stabilization of several proteins, is likely not the only reason for stabilization of LPL by these proteins. This is mainly because lysozyme has a significantly large exposed hydrophobic area. However, the structural properties of proteins that determine their ability to stabilize LPL require further investigation.

The most intriguing observation in this study was the interplay of BSA and heparin in the reversible aggregation / oligomerization of LPL. As shown by ITC, TEM, and RICS measurements, both heparin and albumin alone induced reversible oligomerization of LPL. The LPL aggregates disappeared when both heparin and BSA were present in the incubation solution. This observation suggests that albumin binds to the LPL region involved in heparin-induced oligomerization and that heparin binds to the LPL region that plays a role in albumin-induced aggregation. TEM analysis revealed that albumin-induced LPL aggregates differed from that of heparin-induced LPL helices (**Fig 6**). Unlike heparin-induced higher-order structure helices, albumin-induced aggregates did not have a well-defined regular structure. Despite structural differences, both oligomers were soluble in detergents such as Triton X-100, gum arabic or deoxycholate and dissociated at low LPL concentrations. The solubilization effect of the detergents suggests that the hydrophobic effect may play a role in the formation of both types of oligomers. LFHP was as effective as albumin in dissolving the LPL oligomers induced by heparin, indicating that albumin is the major component of plasma responsible for this effect.

**Fig 9:**
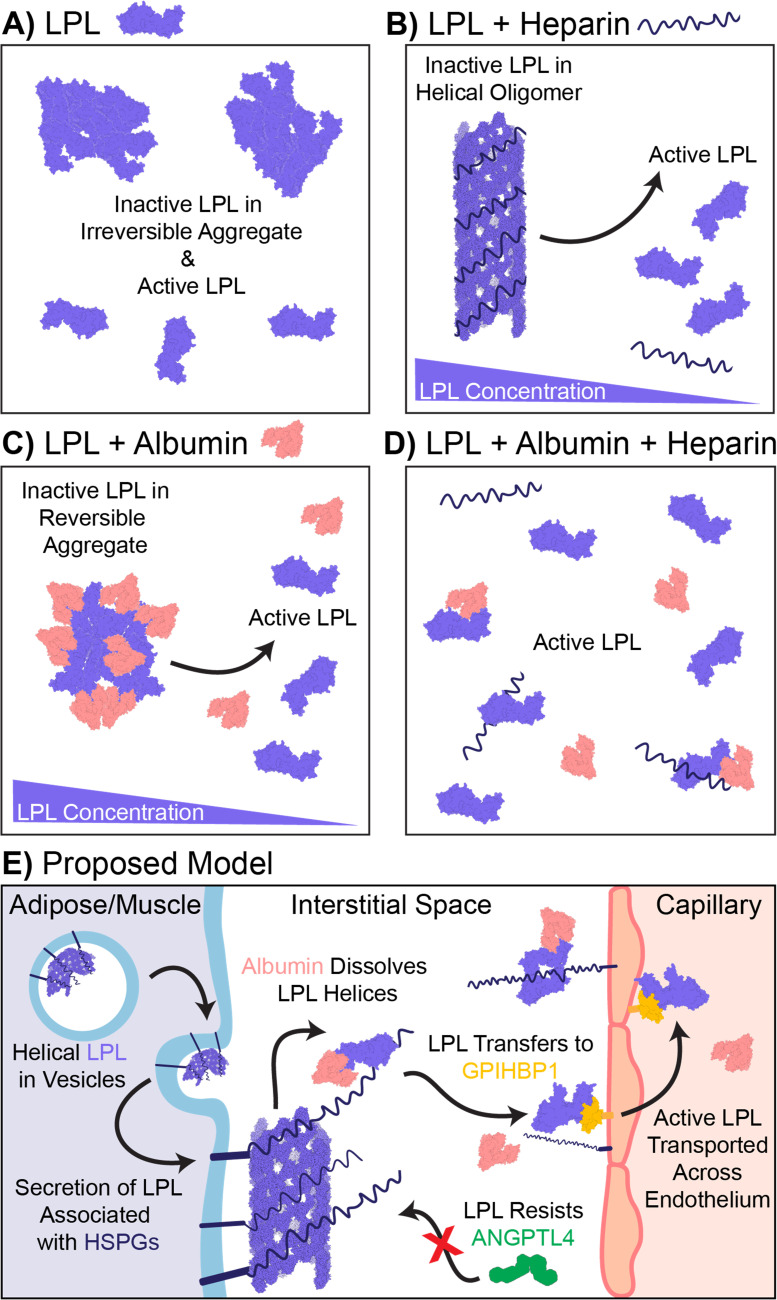
Summarized results and proposed model for oligomerization of LPL. The model describes possible states of LPL according to in vitro investigations.

LPL is mainly synthesized by parenchymal cells of adipose tissue and skeletal muscles, secreted into the interstitial space, and transported by GPIHBP1 to the vascular endothelium. Prior to secretion from cells, LPL has been shown to be transported in intracellular vesicles in which it is complexed with HSPG SDC1 [34] and has acquired filamentous distribution [33]. Upon secretion, the LPL filaments arrive in the interstitial space where vast amounts of albumin and glucose-aminoglycans, including HSPGs, are present. To become enzymatically active, the filaments must dissociate into an active form of LPL. This dissociation can be ensured by the coaction of interstitial albumin and HSPG. Thus, albumin can have a role in creating a suitable environment that turns oligomerized LPL into its active form (**Fig 9**).

It is possible that ANGPTL4 can act on LPL either in the cells that produce them [56], in the subendothelial space right after their secretion [57] or at the luminal side of vascular endothelium [41, 51, 58]. Our experiments show that the ANGPTL4 induced inactivation of LPL occurs faster when both albumin and heparin are present, i.e. in an environment in which LPL is not oligomerized. Both heparin-induced and albumin-induced oligomerization protected LPL from the action of ANGPTL4. It has been shown that nANGPTL4 binds to the lid-domain and the lid-proximal helix of LPL [59]. According to the cryo-EM structure of helical oligomers of LPL, the lid region is involved in the formation LPL helices. Thus, the ANGPTL4 binding region could be occluded in oligomeric LPL, and this might prove to be a fail-safe to protect vesicle-stored LPL in adipose tissue. Inversely, albumin and HSPG are present in the subendothelial space, which might provide a suitable environment for the action of ANGPTL4 on LPL.

Albumin acts as a FFA acceptor *in vivo* during lipolysis and has to be used *in vitro* with substrates that contain long-chain triglycerides to bind released FFAs which would otherwise inhibit LPL. We did not observe any changes to LPL activity when another fatty acid acceptor, cyclodextrin, was used in place of albumin. This indicates that when LPL is in the presence of substrate, the role of albumin is limited to its ability to bind FFAs–and does not rely on a direct physical interaction between LPL and albumin. Further suggesting that the realm of LPL-albumin interactions might be prior to transfer to the vascular endothelium. LPL stabilization has been previously observed when LPL is bound to surfaces of artificial substrate emulsions [46]. It is likely that LPL is also stable on the surface of lipoproteins and is not influenced by albumin or macromolecular crowding of plasma.

A model for describing our observations on the oligomerization of LPL is proposed in **Fig 9**. Depending on the conditions, LPL can appear as an irreversible aggregate, a heparin-induced reversible helical oligomer [33], a reversible albumin-induced aggregate, or in its active form—which could be a properly folded monomer or dimer. The presence of both heparin and albumin leads to dissociation of both reversible type aggregates [42].

In summary, this study suggests that albumin can strongly affect the action and properties of LPL. Albumin may play a role in the decomposition of helical LPL oligomers induced by heparin or HSPGs. The dissociation of LPL oligomers or the avoidance of their formation can be crucial for LPL’s interaction with its ligands as it transits the interstitial space. *In vitro*, both albumin alone and heparin alone promote the concentration-dependent formation of LPL oligomers. These oligomers are reversible and dissociate into catalytically active LPL upon dilution and/or treatment with Triton X-100. Great consideration into LPL concentration and buffer environment should be taken in studies to distinguish between irreversible inactivation or aggregation and reversible LPL oligomer formation, which might affect interactions with various ligands and drugs.

## Supporting information

S1 Fig LPL activity measured in various substrate systems. LPL activity was measured in substrate systems with increasing complexity: soluble fluorescent substrate DGGR < Tributyrin < Intralipid < Isolated triglyceride-rich lipoproteins (CM/VLDL) < Undiluted human plasma. 200 nM LPL was incubated for 15, 45 or 75 min in 20 mM HEPES, 150 mM NaCl, pH 7.4 buffer with 50 mg/ml BSA. Remaining LPL activity was determined with ITC for all substrates except DGGR, where fluorimetry was used instead. A single 5 nM LPL injection was made in the ITC experiments and the final concentration of LPL with DGGR was 10 nM. Results are presented as mean ± SD of three independent measurements and calculated relative to measurements where heparin was added in addition to albumin.
(TIF)

S2 Fig Determining diffusion coefficients of LPL using RICS. **(A)** Estimation of diffusion coefficient (D) for all image sectors acquired with 200 nM LPL, 50 mg/ml BSA and 10 IU/ml heparin. Notice the spread of Ds and that some of the estimates had rather low value, corresponding to images with the larger particles. **(B)** Fit of fluorescence autocorrelation obtained in RICS experiment at 289 Hz scanning speed after analyzing average correlation and filtering the data (see main text for details). **C**—10 nM LPL-ATTO610 incubated in 1 M GuHCl for 2 hours at room temperature. There are only a few bright particles in these conditions (red circle).
(TIF)

S3 Fig Addition of heparin to LPL incubation with albumin. 190 nM LPL with 10 nM LPL-ATTO610 was incubated at room temperature in 20 mM HEPES, 150 mM NaCl, pH 7.4 buffer with 50 mg/ml BSA to mimic conditions used for FCS measurements. After 15 minutes, final concentration of 10 IU/ml heparin or water was added to the mixture. LPL activity expressed as heat rate was measured with ITC at 25°C in 1.31 mM triglycerides human plasma after a 5 nM LPL injection. Data is presented as mean of two independent measurements.
(TIF)

S4 Fig BSA and heparin occasionally form aggregates. **(A)** Aggregates of BSA were not observed when 2 mg/ml BSA was applied to a TEM grid. **(B)** However, aggregates were sometimes observed when 2 mg/ml BSA and 10 IU/ml heparin were mixed together. **(C)** These BSA/heparin aggregates were also seen in a minority of 200 nM LPL with 2 mg/mL BSA and 10 IU/mL heparin micrographs. Scale bars 100 nm. **D**—200 nM LPL in 20 mM HEPES, 150 mM NaCl, pH 7.4 buffer.
(TIF)

S5 Fig Incubation of heparin treated LPL with triton X-100 abolishes formation of LPL helices. When 200 nM of LPL is mixed with 10 IU/ml heparin, the formation of LPL helices was observed (**Fig 6D**). When 200 nM LPL with 10 IU/ml heparin was treated with increasing concentrations of the detergent triton X-100, the LPL helices were progressively dissolved. (**A)**—At 0.005% triton X-100 concentration the beginning of LPL helix dissolution can be observed, with the helices pulling apart (upper panel) or dissolving from one end of the helix (lower panel). **(B)—**At 0.05% triton X-100 LPL helices are no longer observed, although some clumps of LPL are visible. **(C)**—at 0.5% triton X-100 the LPL particles are disperse and do not appear aggregated. Scale bars are 100 nm.
(TIF)
