# Supplementary figures and images for: Combined action of albumin and heparin regulates lipoprotein lipase oligomerization, stability, and ligand interactions
Source: PLoS One. 2023 Apr 12;18(4):e0283358. doi: 10.1371/journal.pone.0283358 (PMC10096250; doi:10.1371/journal.pone.0283358)

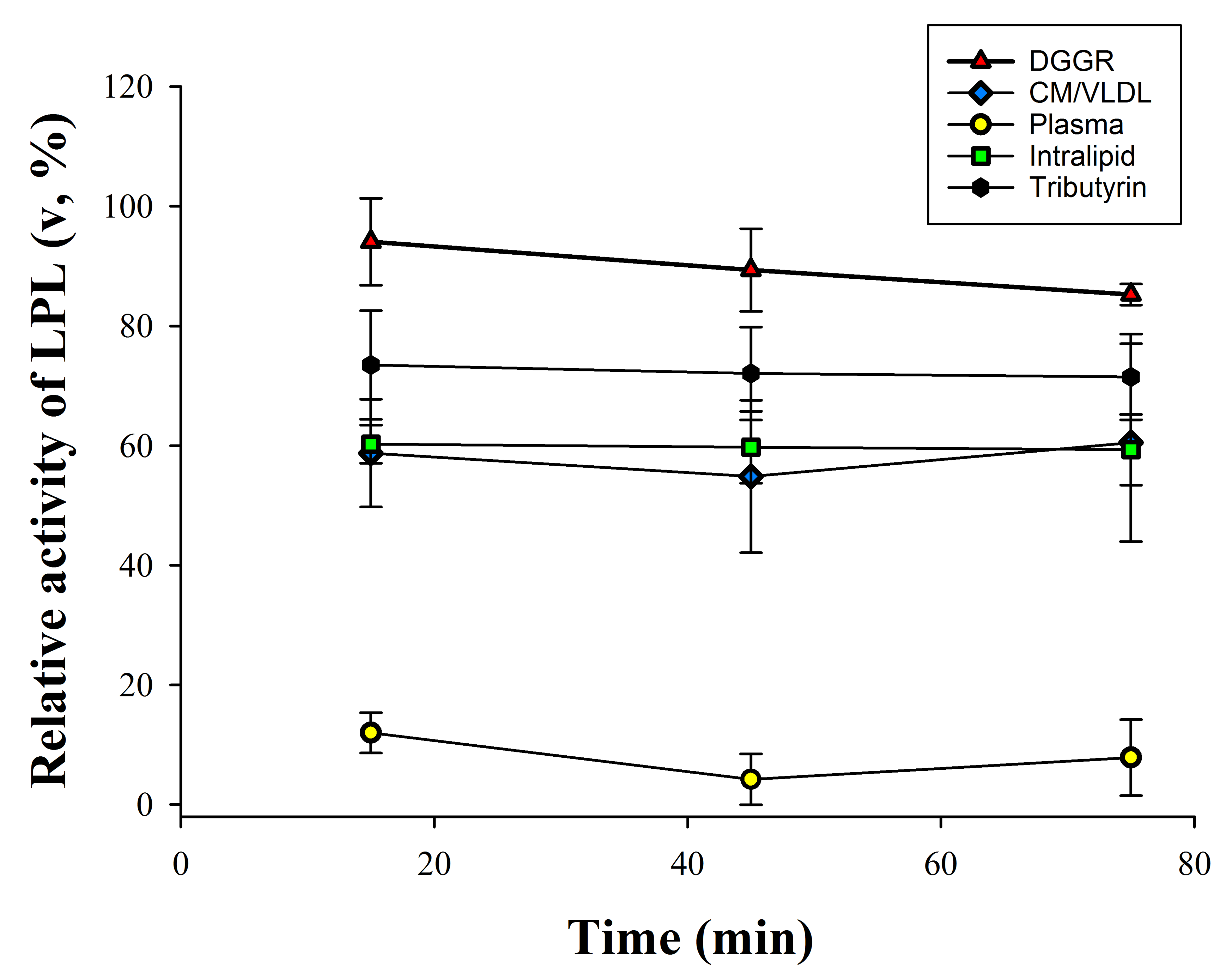

Supplement: S1 Fig — LPL activity was measured in substrate systems with increasing complexity: soluble fluorescent substrate DGGR < Tributyrin < Intralipid < Isolated triglyceride-rich lipoproteins (CM/VLDL) < Undiluted human plasma. 200 nM LPL was incubated for 15, 45 or 75 min in 20 mM HEPES, 150 mM NaCl, pH 7.4 buffer with 50 mg/ml BSA. Remaining LPL activity was determined with ITC for all substrates except DGGR, where fluorimetry was used instead. A single 5 nM LPL injection was made in the ITC experiments and the final concentration of LPL with DGGR was 10 nM. Results are presented as mean ± SD of three independent measurements and calculated relative to measurements where heparin was added in addition to albumin. (TIF) [file pone.0283358.s001.tif]

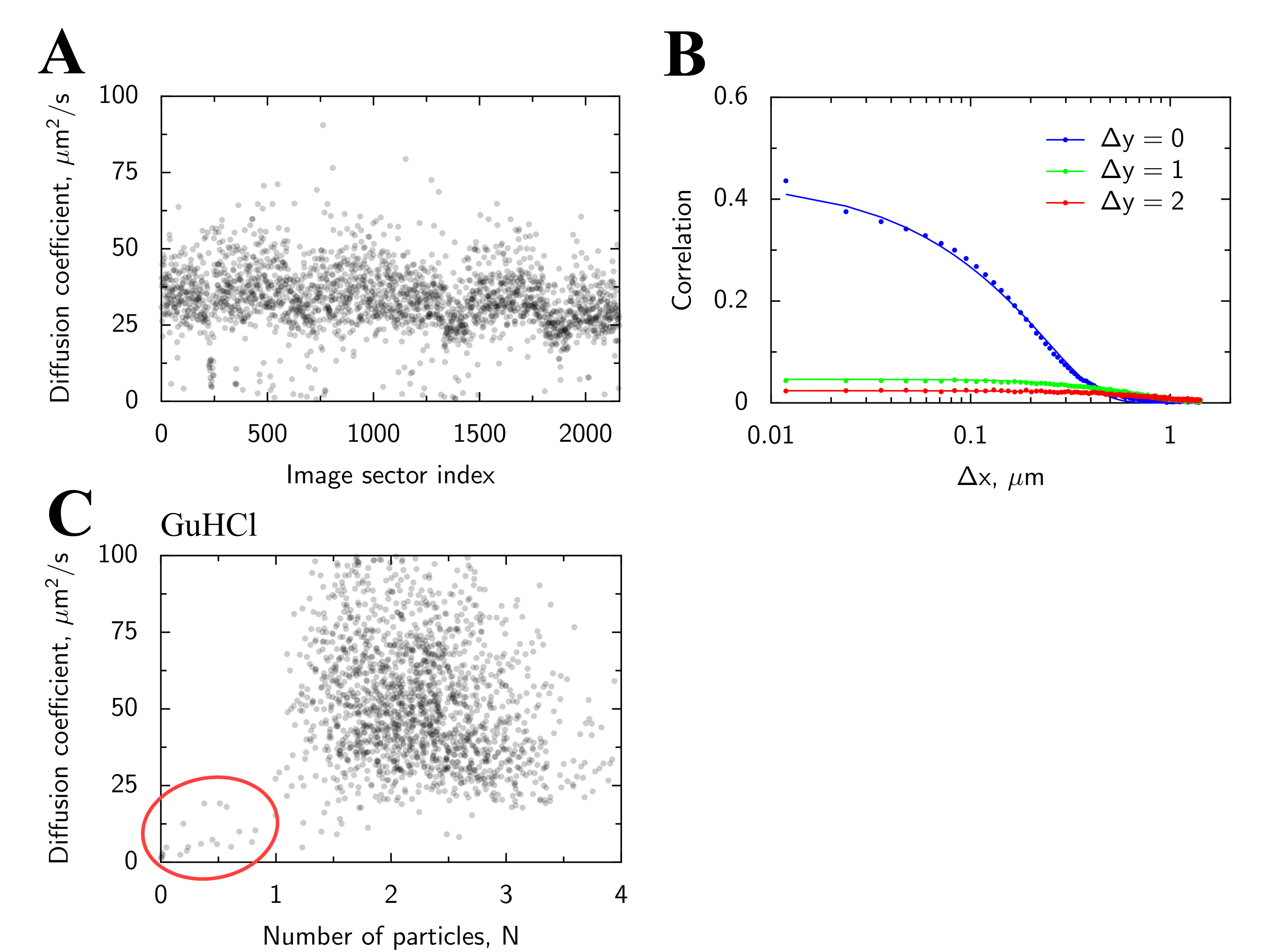

Supplement: S2 Fig — (A) Estimation of diffusion coefficient (D) for all image sectors acquired with 200 nM LPL, 50 mg/ml BSA and 10 IU/ml heparin. Notice the spread of Ds and that some of the estimates had rather low value, corresponding to images with the larger particles. (B) Fit of fluorescence autocorrelation obtained in RICS experiment at 289 Hz scanning speed after analyzing average correlation and filtering the data (see main text for details). C—10 nM LPL-ATTO610 incubated in 1 M GuHCl for 2 hours at room temperature. There are only a few bright particles in these conditions (red circle). (TIF) [file pone.0283358.s002.tif]

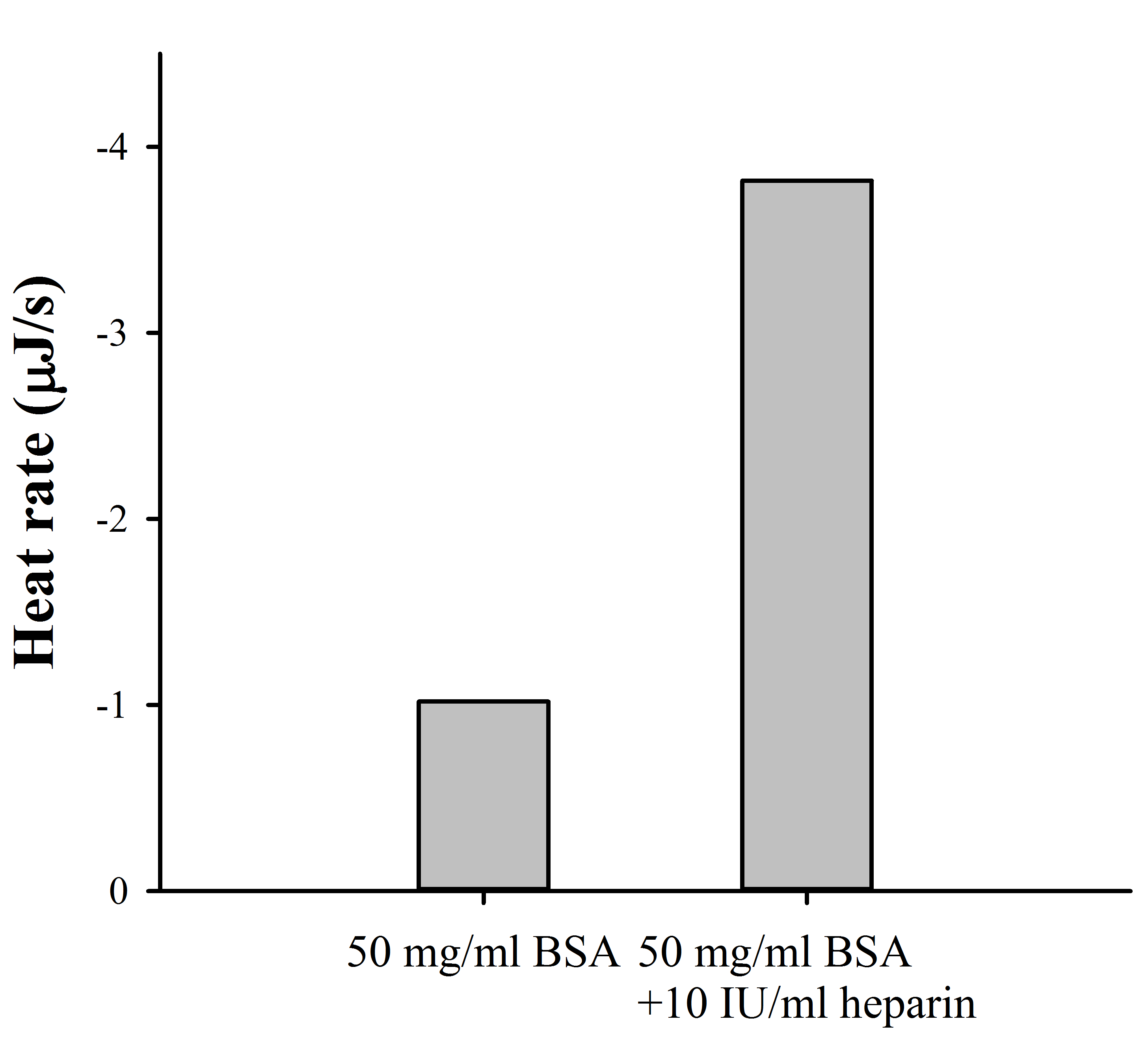

Supplement: S3 Fig — 190 nM LPL with 10 nM LPL-ATTO610 was incubated at room temperature in 20 mM HEPES, 150 mM NaCl, pH 7.4 buffer with 50 mg/ml BSA to mimic conditions used for FCS measurements. After 15 minutes, final concentration of 10 IU/ml heparin or water was added to the mixture. LPL activity expressed as heat rate was measured with ITC at 25°C in 1.31 mM triglycerides human plasma after a 5 nM LPL injection. Data is presented as mean of two independent measurements. (TIF) [file pone.0283358.s003.tif]

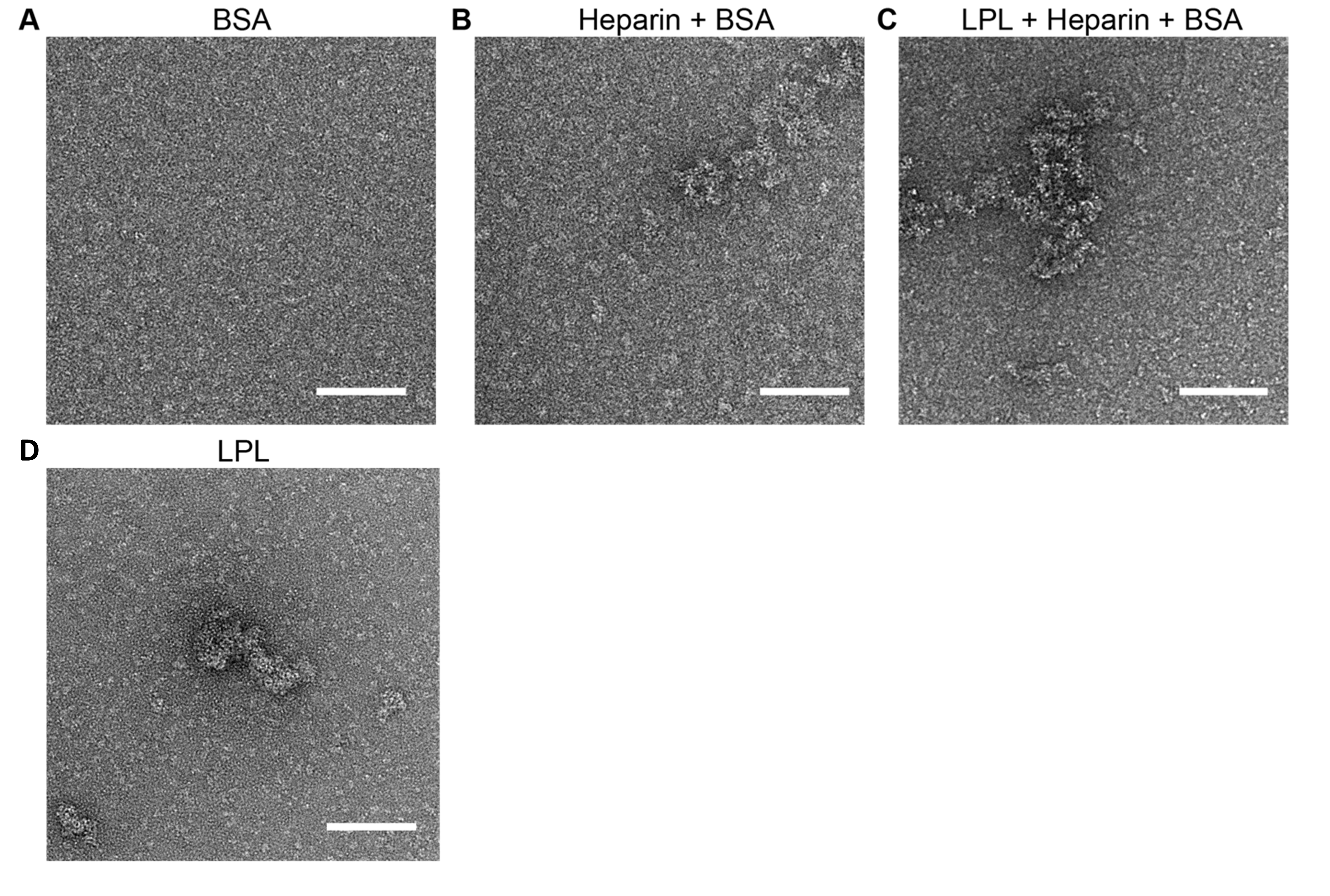

Supplement: S4 Fig — (A) Aggregates of BSA were not observed when 2 mg/ml BSA was applied to a TEM grid. (B) However, aggregates were sometimes observed when 2 mg/ml BSA and 10 IU/ml heparin were mixed together. (C) These BSA/heparin aggregates were also seen in a minority of 200 nM LPL with 2 mg/mL BSA and 10 IU/mL heparin micrographs. Scale bars 100 nm. D—200 nM LPL in 20 mM HEPES, 150 mM NaCl, pH 7.4 buffer. (TIF) [file pone.0283358.s004.tif]

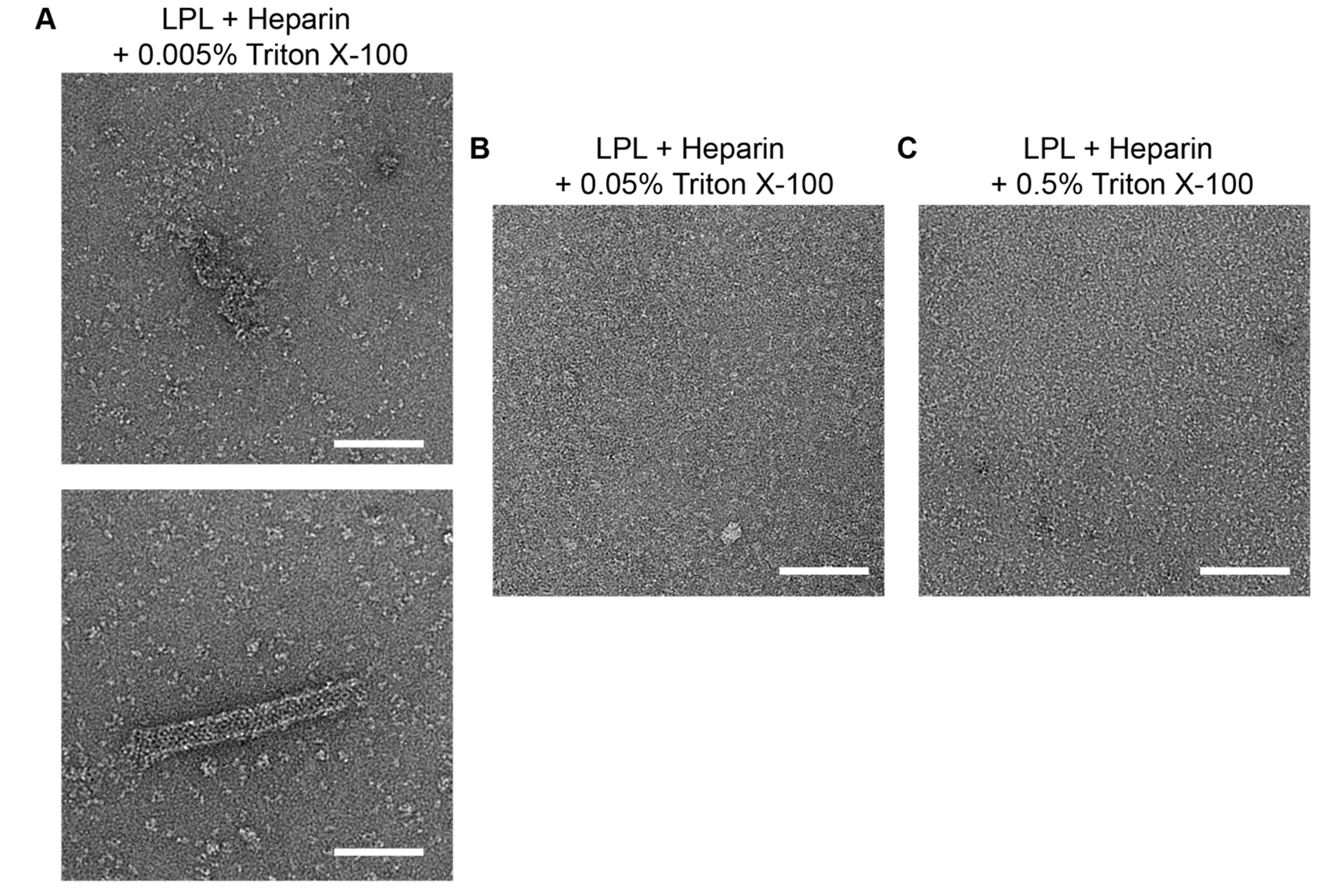

Supplement: S5 Fig — When 200 nM of LPL is mixed with 10 IU/ml heparin, the formation of LPL helices was observed (Fig 6D). When 200 nM LPL with 10 IU/ml heparin was treated with increasing concentrations of the detergent triton X-100, the LPL helices were progressively dissolved. (A)—At 0.005% triton X-100 concentration the beginning of LPL helix dissolution can be observed, with the helices pulling apart (upper panel) or dissolving from one end of the helix (lower panel). (B)—At 0.05% triton X-100 LPL helices are no longer observed, although some clumps of LPL are visible. (C)—at 0.5% triton X-100 the LPL particles are disperse and do not appear aggregated. Scale bars are 100 nm. (TIF) [file pone.0283358.s005.tif]
